# Synthesis of novel alkynyl imidazopyridinyl selenides: copper-catalyzed tandem selenation of selenium with 2-arylimidazo[1,2-*a*]pyridines and terminal alkynes

**DOI:** 10.3762/bjoc.18.87

**Published:** 2022-07-19

**Authors:** Mio Matsumura, Kaho Tsukada, Kiwa Sugimoto, Yuki Murata, Shuji Yasuike

**Affiliations:** 1 School of Pharmaceutical Sciences, Aichi Gakuin University, 1-100 Kusumoto-cho, Chikusa-ku, Nagoya 464-8650, Japan

**Keywords:** alkynyl imidazopyridinyl selenide, copper catalyst, imidazo[1,2-*a*]pyridine, selenium, tandem reaction, terminal alkyne

## Abstract

Alkynyl selenides have attracted considerable research interest recently, owing to their applications in the biological and pharmaceutical fields. The Cu-catalyzed tandem reaction for the synthesis of novel alkynyl imidazopyridinyl selenides is presented. A one-pot synthesis route afforded alkynyl imidazopyridinyl selenides in moderate to good yields. This was achieved by a two-step reaction between terminal alkynes and diimidazopyridinyl diselenides, generated from imidazo[1,2-*a*]pyridines and Se powder, using 10 mol % of CuI and 1,10-phenanthroline as the catalytic system under aerobic conditions. The C(sp^2^)–Se and C(sp)–Se bond-formation reaction can be performed in one-pot by using inexpensive and easy to handle Se powder as the Se source. The reaction proceeded with terminal alkynes having various substitutions, such as aryl, vinyl, and alkyl groups. The obtained alkynyl imidazopyridinyl selenide was found to undergo nucleophilic substitution reaction on Se atom using organolithium reagents and 1,3-dipolar azide–alkyne cycloaddition based on the alkyne moiety.

## Introduction

**Figure 1:**
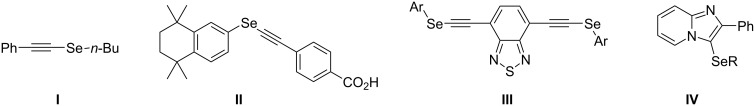
Biologically active selenides having alkynyl or imidazopyridinyl groups.

Imidazo[1,2-*a*]pyridines are important heterocycles that serve as key functional groups in many biologically active substances and pharmaceuticals, such as zolpidem, alpidem, and GSK812397 [1–3]. Therefore, the development of multiple chemical modification methods, at the 3-position of the imidazo[1,2-*a*]pyridine skeleton, for the synthesis of 3-substituted-imidazo[1,2-*a*]pyridines with potential bioactivity, has been reported [4–7]. Similarly, organoselenium compounds have also received increased attention in recent years due to their promising applications as bioactive substances in drug discovery [8]. Among these compounds, alkynyl selenides have attracted interest in the last two decades owing to their applicability in biological and pharmaceutical fields [9–10]. For example, butyl (2-phenylethynyl)selenide (**I**, Figure 1) has an antinociceptive effect on the formalin test in mice [11], selanyl acetylenic retinoids **II** show an RAR agonist activity [12], and bis-alkynylselenides **III** exhibits a binding interaction between calf-thymus DNA and human serum albumin [13]. Moreover, imidazopyridine derivatives **IV** with selanyl groups, 3-aryl- or alkylselanylimidazo[1,2-*a*]pyridines, were reported to act as potential antioxidants and showed antiproliferative activity [14–15]. However, there is no reported example of the synthesis of alkynyl imidazopyridinyl selenides.

The Se–C bond-formation reaction using transition metals such as Pd, Ru, Ni, Fe, and Cu as catalysts is one of the most powerful synthetic tools for preparing organoselenium compounds [16–18]. Diselenides (RSeSeR) and selanyl halides (RSeX) have been widely used as Se sources in these reactions. However, they have poor commercial availability and require complicated synthetic routes. Therefore, an alternative approach using the commercially available and easy-to-handle Se powder has attracted significant attention [15,19–26]. This method involves a transition-metal-catalyzed one-pot, three-component reaction in which two functional groups are simultaneously introduced on the Se atom via double selenation. As examples, the following Cu-catalyzed one-pot reactions have been reported for the synthesis of unsymmetrical selenides with imidazopyridinyl groups substituted at position 3. Guo and Li et al. reported the reaction of Se powder with imidazopyridine and aryl iodides in the presence of KOH (2 equiv) as base using Cu(OAc)_2_ catalyst to form aryl imidazopyridinyl selenides [15]. Guo, Han and Ma et al. also performed the synthesis of aryl imidazopyridinyl selenides in the presence of Ag_2_CO_3_ (2 equiv) and Cs_2_CO_3_ (2 equiv) using the CuI/1,10-phenanthroline catalytic system by replacing the aryl group donor with arylboronic acids [23]. Zhou et al. reported the reaction of Se powder with imidazopyridine and aryl iodides or alkyl halides in the presence of Na_2_CO_3_ (2 equiv) using the NiBr_2_/2,2-bipyridine system to give aryl or alkyl imidazopyridinyl selenides [24]. In these reactions, aryl iodides, arylboronic acids, and alkyl halides are coupled with Se powder to form diaryl or dialkyl diselenides, followed by C–H selenation with imidazopyridines to form the corresponding compounds. We also reported the one-pot two-step reaction of Se powder with imidazopyridine and triarylbismuthines using the CuI/1,10-phenanthroline catalytic system without bases, which formed similar selenides [25]. In this reaction, unlike the former, bis(imidazo[1,2-*a*]pyridin-3-yl) diselenides are generated through C–H selenation at the 3-position of 2-arylimidazopyridines with Se powder, followed by the cross-coupling reaction between diselenides and triarylbismuthines. These one-pot reactions are limited to C(sp^2^)–Se–C(sp^2^) or C(sp^2^)–Se–C(sp^3^) bond-formation reactions. However, C(sp^2^)–Se–C(sp) bond formation reactions using the imidazopyridines and alkyne derivatives have not been reported to date. Based on previous reports and our ongoing investigation of the synthesis of unsymmetrical selenides with an imidazo[1,2-*a*]pyridine ring, this study focused on the Cu-catalyzed one-pot C(sp^2^)–Se and C(sp)–Se bond formation for the synthesis of novel alkynyl imidazopyridinyl selenides using Se powder, 2-arylimidazo[1,2-*a*]pyridines, and terminal alkynes.

## Results and Discussion

### Synthesis of alkynyl imidazopyridinyl selenides

A Cu-catalyzed cross-coupling reaction using benzene ring substituted diaryl diselenides with terminal alkynes in the presence of bases is effective for synthesizing aryl alkynyl selenides [27–31]. We previously reported a simple method for the synthesis of bis(2-arylimidazo[1,2-*a*]pyridin-3-yl) diselenides using a Cu-catalyzed C–H selenation at the 3-position of 2-arylimidazo[1,2-*a*]pyridines with Se powder [32]. Initially, the reaction of bis(2-phenylimidazo[1,2-*a*]pyridin-3-yl) diselenide (**2a**), generated from Se powder and 2-phenylimidazo[1,2-*a*]pyridine (**1a**), with phenylacetylene (**3a**) was used as a model reaction to determine a suitable base and an equivalent number of reagents (Table 1). The key intermediate **2a** was prepared in situ from **1a** (0.5 mmol) and Se powder (0.5 mmol) in the presence of 10 mol % of CuI and 1,10-phenanthroline at 130 °C in DMSO under aerobic conditions without bases using the method reported previously [25,32]. The reaction mixture was then treated with phenylacetylene (**3a**, 0.5 mmol) and various bases at room temperature. The formation of **4aa** was confirmed by thin-layer chromatography. The use of various bases (2 equiv) such as K_2_CO_3_, Na_2_CO_3_, and triethylamine resulted in the formation of the expected alkynyl imidazopyridinyl selenide **4aa** in moderate to good yields (Table 1, entries 1–8). Among them, Na_2_CO_3_ was identified as the optimal base in terms of yield of product **4aa** and reaction time (entry 5 in Table 1). Notably, during the one-pot reaction, both selanyl groups from the diselenide transferred to the product **4aa**. When no base was added or the amount of the base was reduced, the yield of **4aa** decreased significantly (Table 1, entries 9 and 10). Moreover, increasing the amount of the alkyne **1a** or Na_2_CO_3_ did not affect the progress of the reaction (Table 1, entries 5, 11, and 12). Consequently, the optimal result was obtained when diselenide **2a** was treated with equal amounts of alkyne **1a** and two equivalents of Na_2_CO_3_ under aerobic conditions at room temperature (Table 1, entry 5). The reaction was also attempted by mixing the three components (Se powder, **1a**, and **3a**) in the presence of Na_2_CO_3_ at 130 °C, but unfortunately it did not proceed and gave a complex mixture (Table 1, entry 13).

**Table 1:** One-pot reaction of Se powder with **1a** and **3a**^a^.

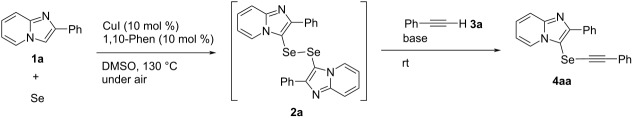				

Entry	Alkyne **3a** (equiv)	Base (equiv)	Time (h)	Yield of **4aa** (%)^b^

1	1	K_2_CO_3_ (2)	3	60
2	1	K_3_PO_4_ (2)	3	34
3	1	KOH (2)	3	20
4	1	KO*t-*Bu (2)	24	30
5	1	Na_2_CO_3_ (2)	2	74
6	1	Cs_2_CO_3_ (2)	2	36
7	1	NaHCO_3_ (2)	4	42
8	1	Et_3_N (2)	3	73
9	1	Na_2_CO_3_ (1)	3	42
10	1	–	24	20
11	2	Na_2_CO_3_ (4)	2	73
12	1	Na_2_CO_3_ (3)	2	69
13^c^	1	Na_2_CO_3_ (2)	1	–

^a^Conditions: **1a** (0.5 mmol), Se (0.5 mmol), **3a** (0.5 mmol), CuI (0.05 mmol), 1,10-phenanthroline (0.05 mmol), DMSO (3 mL); ^b^isolated yield; ^c^simultaneously added all reagents of **1a**, Se, **3a**, and Na_2_CO_3_ at 130 °C.

**Figure 2:**
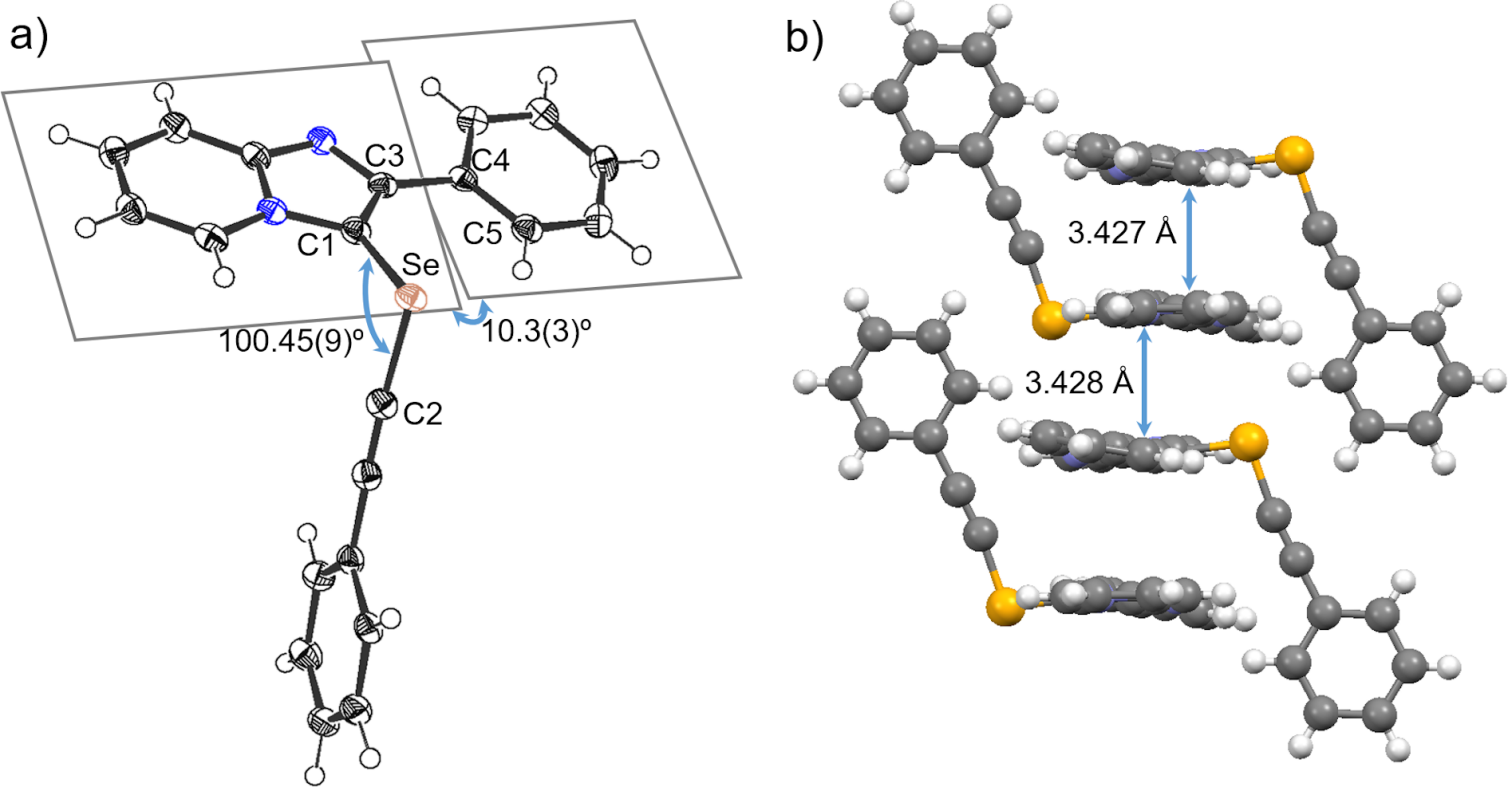
(a) ORTEP drawing of **4aa** and (b) its stacking structure.

Single crystals suitable for X-ray analysis of **4aa** were obtained by repeated recrystallization from dichloromethane/hexane as solvent. Figure 2a shows the crystal structure of **4aa**, revealing that the ethynylselanyl group is located at the 3-position of the imidazo[1,2-*a*]pyridine core, and the bond angle of C1–Se–C2 was 100.45°. The imidazo[1,2-*a*]pyridine plane and the phenyl ring were observed to be slightly twisted, with a C2–C3–C4–C5 torsion angle of 10.28°. 2-Phenylimidazo[1,2-*a*]pyridine moieties of neighboring molecules were arranged into slipped-parallel π-stacks with head-to-tail or head-to-head orientations. The distances between parallel mean planes were 3.427 and 3.428 Å (Figure 2b).

To demonstrate the efficiency and to broaden the scope of the above-mentioned one-pot, two-step protocol, the reactions of 2-arylimidazopyridines **1** (1 mmol), Se powder (1 mmol), and terminal alkynes **3** (1 mmol) in the presence of Na_2_CO_3_ (2 mmol) were investigated under the optimized conditions. This method involved forming the diselenides, followed by the addition of terminal alkynes and Na_2_CO_3_ to the same reaction flask at room temperature. The reaction of imidazopyridine **1a** and Se powder with various terminal alkynes **3b**–**e** containing aryl groups yielded the corresponding arylalkynyl imidazopyridinyl selenides **4ab**–**ae**, respectively, in 41–57% yields (Table 2, entries 1–4). The electronic nature of the substituents in the arylalkynes **3** did not affect the outcome of the reaction. For terminal alkynes **3f** and **3g** with a heteroaryl group such as thiophene (Table 2, entry 5) and the vinyl group (Table 2, entry 6), the coupling products **4af** and **4ag** were also isolated with yields of 61% and 71%, respectively. Conversely, 1-hexyne **4h** with a butyl moiety as the alkyl group formed **4ah** with a lower yield (see Table 2, entry 7) than those observed for other terminal alkynes.

**Table 2:** One-pot two-step synthesis of alkynyl imidazopyridinyl selenides **4**^a^.

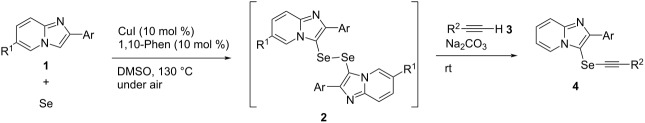			

Entry	Product **4**	Yield^b^	Reaction time^c^

1	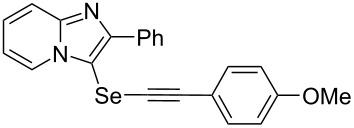 **4ab**	57%	2 h (2 h)
2	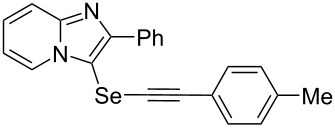 **4ac**	51%	2 h (3 h)
3	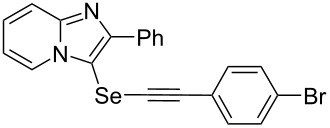 **4ad**	41%	2 h (2 h)
4	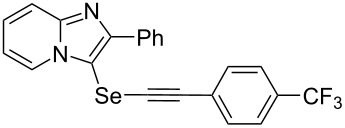 **4ae**	48%	2 h (2 h)
5	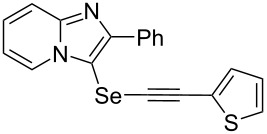 **4af**	61%	2 h (3 h)
6	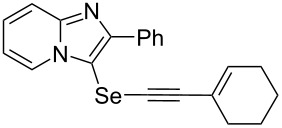 **4ag**	71%	2 h (5 h)
7	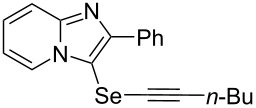 **4ah**	31%	2 h (21 h)
8	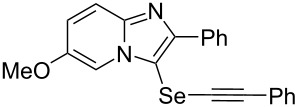 **4ba**	69%	2 h (2 h)
9	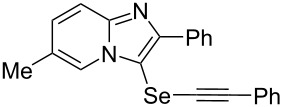 **4ca**	58%	2 h (2 h)
10	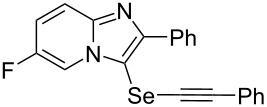 **4da**	77%	2 h (2 h)
11	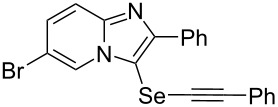 **4ea**	41%	3 h (3 h)
12	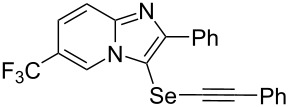 **4fa**	0%	24 h
13	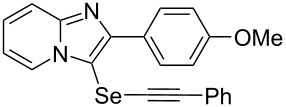 **4ga**	68%	2 h (2 h)
14	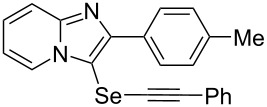 **4ha**	61%	2 h (2 h)
15	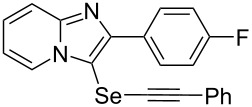 **4ia**	69%	3 h (2 h)
16	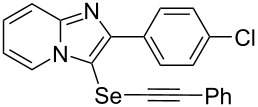 **4ja**	66%	2 h (3 h)
17	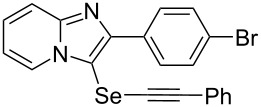 **4ka**	72%	2 h (2 h)
18	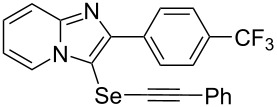 **4la**	51%	3 h (2 h)
19	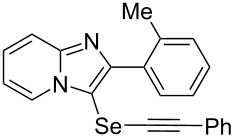 **4ma**	59%	2 h (2 h)

^a^Conditions: **1** (1 mmol), **3** (1 mmol), Se (1 mmol), CuI (0.1 mmol), 1,10-phenanthroline (0.1 mmol), Na_2_CO_3_ (2 mmol), DMSO (3 mL); ^b^isolated yields; ^c^the reaction time for the synthesis of diselenides **2** is provided, followed by the time for alkynyl imidazopyridinyl selenides **4** in parentheses.

Next, the reaction of 2-phenylimidazopyridines **1b**–**e** with electron-donating or halogen groups at the 6-position of the imidazopyridine ring were tested and gave the desired products **4ba**–**ea** in fair to good yields (Table 2, entries 8–11). Conversely, imidazopyridine **1f** with an electron-withdrawing trifluoromethyl group did not yield the corresponding product **4fa**, because the diselenide **2f** could not be generated (Table 2, entry 12). The reaction not only proceeded for the 6-substituted 2-phenylimidazopyridines but also for 2-arylimidazopyridines **1g**–**m** with a substituent on the phenyl group at the 2-position of the imidazopyridine ring, and the coupling products **4ga**–**ma** were obtained in 51–72% yields (Table 2, entries 13–19).

**Scheme 1:**
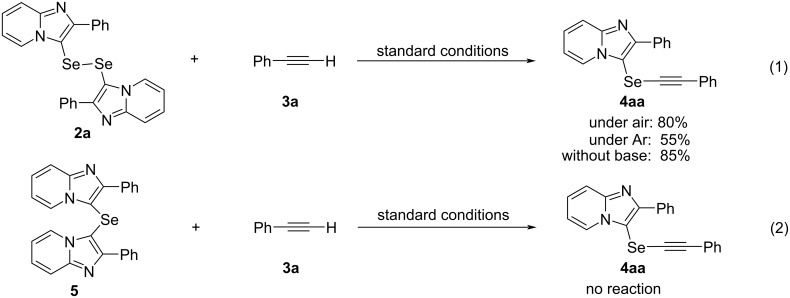
Control reactions.

We also performed control experiments to investigate the reaction pathway and mechanism. The reaction of the isolated diselenide **2a** with phenylacetylene (**3a**) under the standard conditions afforded the desired product **4aa** in 80% yield. On the other hand, under an argon atmosphere, the reaction yield decreased by approximately half. This reaction, without Na_2_CO_3_ as base, also afforded **4aa** in 85% yield (Scheme 1, reaction 1). Although bis(imidazo[1,2-*a*]pyridin-3-yl)monoselenides **5** could form in situ [32], the reaction of **5** with acetylene **3a** under the standard conditions did not proceed (Scheme 1, reaction 2).

**Figure 3:**
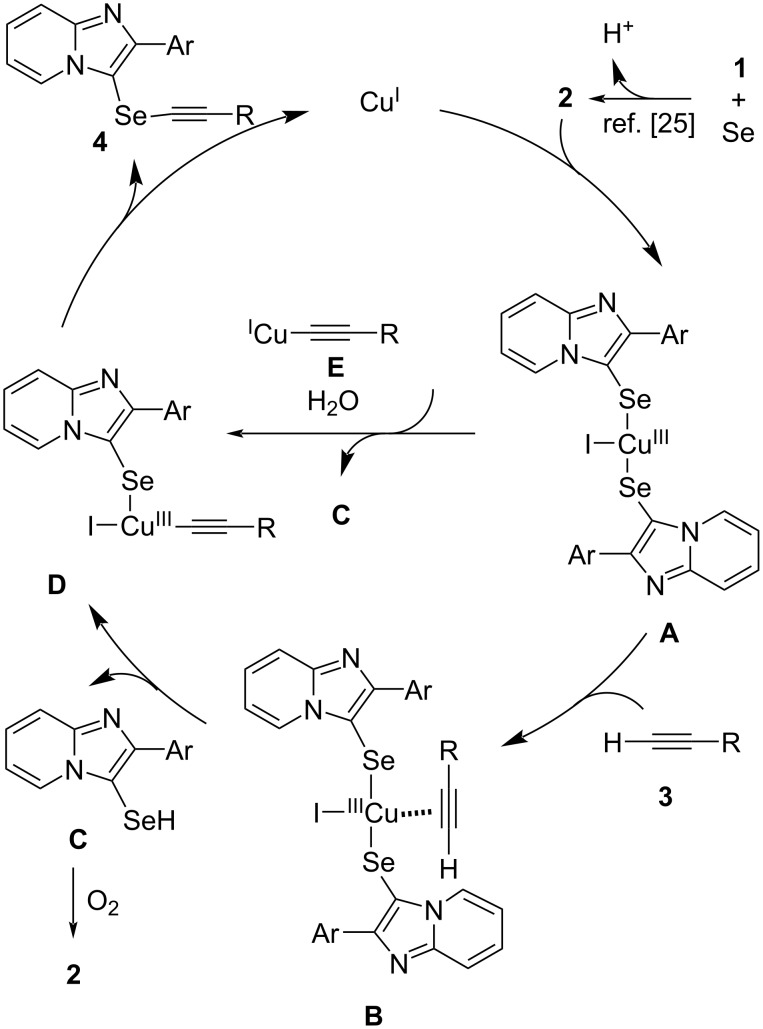
Proposed mechanism.

The reaction mechanism for this coupling reaction is presently unclear. We propose that the reaction mechanism may be similar to that of the C(sp)–Se bond formation of terminal alkynes with diaryl diselenides reported by Stieler and Schneider [31]. Based on this report and the above control experiments, a plausible selenation reaction mechanism is shown in Figure 3. The first step of the reaction involves the generation of intermediate **A** by the oxidative addition of the Cu(I) catalyst to the diselenide **2**. The terminal alkyne coordinates with intermediate **A** to form a π-complex **B**, and a ligand exchange reaction from **B** occurs to produce intermediate **D**, together with the elimination of selenol **C**. The selenol **C** is oxidized to diselenide **2**. Finally, the intermediate **D** undergoes a reductive elimination to form the desired product **4**, with the regeneration of Cu(I). As an alternative route, we also surmise that Cu–acetylide **E** attacks intermediate **A** to produce intermediate **D**. Although this reaction was performed under aerobic conditions, Glaser-type homocoupling of terminal alkynes did not occur, and no diynes were observed as byproducts. Moreover, the reaction of diselenides **2a** with **3a** without a base afforded the corresponding product **4aa** in good yield (Scheme 1, reaction 1). Therefore, it was concluded that this reaction proceeds predominantly via the intermediate **B** route. The base appears to trap the protons generated during the first step involving the derivation of diselenide **2** from imidazopyridine **1** and Se powder.

### Transformation from alkynyl imidazopyridinyl selenides

**Scheme 2:**
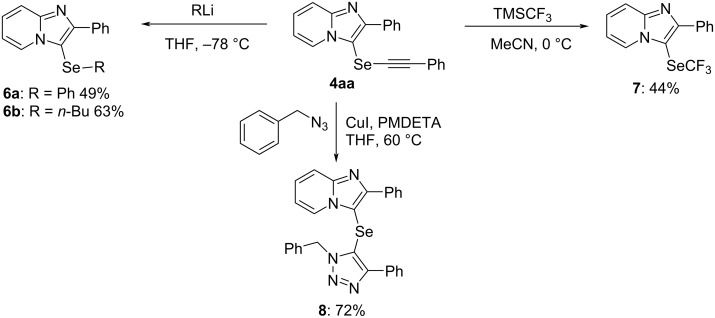
Transformation from **4aa**.

To investigate the chemical reactivity of the alkynyl imidazopyridinyl selenides, several reactions were performed (Scheme 2). Treatment of **4aa** with two equivalents of phenyllithium in THF at −78 °C led to a nucleophilic substitution reaction with the elimination of the ethynyl group to form the desired phenylimidazopyridinyl selenide **6a** in 49% yield. In the reaction with *n*-butyllithium, alkyl derivative **6b** was isolated in the same way. The reaction of **4aa** with the Ruppert–Prakash reagent (TMSCF_3_) in the presence of Cs_2_CO_3_ as base in MeCN at 0 °C gave product **7** with a trifluoromethyl group. Stefani et al. reported the 1,3-dipolar azide–alkyne cycloaddition (AAC) of organotellanyl alkynes with organic azides in the presence of a copper reagent to form 5-organotellanyl-1,2,3-triazoles [33]. Based on these findings, we examined the reaction of Cu-mediated AAC. The reaction of **4aa** with benzyl azide in the presence of one equivalent of CuI and pentamethyldiethylenetriamine (PMDETA) in THF at 60 °C gave the desired 5-selanyl-1,2,3-triazole **8** in 72% yield. This reaction yielded a single product, and the regiochemistry of 5-selanyltriazole **8** was confirmed by single crystal X-ray analysis (see Supporting Information File 1). The reaction performed using 10 mol % of CuI and PMDETA as catalytic system afforded only a small amount of product **8** (12%).

Based on the results obtained in this study, the synthesis route still has some limitations such as the yields of alkynyl imidazopyridinyl selenides and the scope of substrates. Nevertheless, the scope of future research includes the application of this synthesis route using other heterocycles, and the investigation of the biological activity of the compounds obtained via this synthesis route.

## Conclusion

In this study, the synthesis route for novel alkynyl imidazopyridinyl selenides using the Cu-catalyzed one-pot reaction of Se powder with imidazo[1,2-*a*]pyridines and terminal alkynes was developed. A variety of desired compounds was synthesized using a simple operation that can be performed under aerobic conditions. Moreover, the results showed that the obtained compounds underwent nucleophilic substitution reactions involving the elimination of the alkyne moiety on Se atoms to form aryl or alkyl imidazopyridinyl selenides and regioselective 1,3-dipolar azide–alkyne cycloaddition to form 5-selanyl-1,2,3-triazole. The investigation of the biological activity of the compounds obtained in this study and the application of this synthesis route using other heterocycles, instead of imidazopyridine, are currently underway in our laboratory.

## Experimental

### General procedure for the synthesis of alkynyl imidazopyridinyl selenides

A solution of 2-phenylimidazo[1,2-*a*]pyridine (**1a**, 1.0 mmol), selenium powder (79 mg, 1.0 mmol, 1 equiv), CuI (14 mg, 0.1 mmol, 10 mol %) and 1,10-phenanthoroline (18 mg, 0.1 mmol, 10 mol %) in DMSO (3 mL) was heated at 130 °C under air atmosphere. After the reaction was completed, the mixture was allowed to cool to room temperature. Then, the alkyne (**3**, 1.0 mmol, 1 equiv) and Na_2_CO_3_ (212 mg, 2.0 mmol, 2 equiv) were added and the mixture was stirred at room temperature. After the reaction was completed, the reaction mixture was diluted with CH_2_Cl_2_ (30 mL) and 5% aqueous ammonia (30 mL) at 0 °C. The phases were separated and the aqueous layer was extracted with CH_2_Cl_2_ (20 mL × 2). The combined organic layer was washed with 5% aqueous ammonia (30 mL × 3), dried over anhydrous magnesium sulfate, filtered, and concentrated under reduced pressure. The residue was purified by column chromatography using hexane/AcOEt as eluent to give the desired products **4aa**–**ma**.

## Supporting Information

File 1 Characterization data of all new compounds, synthetic procedures for compounds **6**–**8**, X-ray crystallography details, and copies of spectra.

File 2 X-ray crystal structure of **4aa**.

File 3 X-ray crystal structure of **8**.
