# Supplementary material for: Synthesis of novel alkynyl imidazopyridinyl selenides: copper-catalyzed tandem selenation of selenium with 2-arylimidazo[1,2-a]pyridines and terminal alkynes
Source: Beilstein J Org Chem. 2022 Jul 19;18:863–71. doi: 10.3762/bjoc.18.87 (PMC9344556; doi:10.3762/bjoc.18.87)

BEILSTEIN JOURNAL OF ORGANIC CHEMISTRY

Supporting Information

for

Synthesis of novel alkynyl imidazopyridinyl selenides: copper-catalyzed tandem selenation of selenium with 2-arylimidazo[1,2-*a*]pyridines and terminal alkynes

Mio Matsumura, Kaho Tsukada, Kiwa Sugimoto, Yuki Murata and Shuji Yasuike

Beilstein J. Org. Chem. **2022,** *18,* 863–871. doi:10.3762/bjoc.18.87

Characterization data of all new compounds, synthetic procedures for compounds 6–8, X-ray crystallography details, and copies of spectra

License and Terms: This is a supporting information file under the terms of the Creative Commons Attribution License (https://creativecommons.org/licenses/by/4.0). Please note that the reuse, redistribution and reproduction in particular requires that the author(s) and source are credited and that individual graphics may be subject to special legal provisions.
The license is subject to the *Beilstein Journal of Organic Chemistry* terms and conditions: (https://www.beilstein-journals.org/bjoc/terms)

Table of contents

1. General information....................S2

2. Experimental details and characterization data....................S2

3. Single crystal X-ray diffraction experiment S11

4. References S12

5. NMR spectra of novel compounds S13

1. General information

Melting points were taken on a Yanagimoto micro melting point hot-stage apparatus (MP-S3) and are not corrected. ^{1}H NMR (400 MHz, TMS: δ = 0.00 ppm as an internal standard), ^{13}C NMR (100 MHz, $CDCl_3$: δ = 77.00 or benzene-d_6: δ = 128.06 ppm as an internal standard), ^{19}F NMR (376 MHz, benzotrifluoride; δ = −64.0 ppm as an external standard) and ^{77}Se NMR (76 MHz, diphenyldiselenide; δ = 463.15 ppm as an external standard) spectra were recorded on JEOL ECZ-400S spectrometer (JEOL Ltd., Tokyo, Japan) in $CDCl_3$ or benzene-d_6. GC–MS (EI) spectra were recorded on an Agilent 5977E Diff-SST MSD-230V spectrometer (Agilent Technologies Japan, Ltd., Tokyo, Japan). ESI mass spectra were measured on an Agilent Technologies 6230 LC/TOF mass spectrometer (Agilent Technologies Japan, Ltd., Tokyo, Japan). IR spectra were recorded on a SHIMADZU FTIR-8400S spectrometer (SHIMADZU CORPORATION, Kyoto, Japan) and are reported in frequency of absorption (cm^{-1}). Only selected IR peaks are reported. The X-ray diffraction measurements were carried out using an XtaLAB Synergy, Single source at home/near, HyPix3000 diffractometer (Rigaku, Tokyo, Japan). All chromatographic separations were accomplished with Silica Gel 60N (Kanto Chemical Co., Inc., Tokyo, Japan). Thin-layer chromatography (TLC) was performed with Macherey–Nagel precoated TLC plates Sil G25 UV_{254}. Most of the reagents were used without further purification unless otherwise specified.

Various imidazo[1,2-*a*]pyridine derivatives (**1a**–**m**) were prepared according to the reported procedures [1]. The spectroscopic data of known selanylimidazo[1,2-*a*]pyridines **5a** [1], **5b** [2], and **6** [3] are in accordance with the literature.

2. Experimental details and characterization data

Preparation and characterization of alkynyl imidazopyridinyl selenides

Compounds 4aa–**ma** were prepared following the general procedure provided in the Experimental.

2-Phenyl-3-[(2-phenylethynyl)selanyl]imidazo[1,2-*a*]pyridine (**4aa**)

Colorless prisms (275 mg, 74%), m.p. 84–86°C (CH_2Cl_2/hexane/Et_2O). ^{1}H-NMR (400 MHz, $CDCl_3$) δ (ppm): 8.58 (d, *J* = 6.7 Hz, 1H), 8.13 (dt, *J* = 8.2, 1.4 Hz, 2H), 7.75 (d, *J* = 8.7 Hz, 1H), 7.52 (t, *J* = 8.2 Hz, 2H), 7.43 (tt, *J* = 7.2, 1.2 Hz, 1H), 7.39–7.34 (m, 3H), 7.29–7.23 (m, 3H), 7.02 (td, *J* = 6.9, 1.4 Hz, 1H). ^{13}C-NMR (100 MHz, $CDCl_3$) δ (ppm): 150.2 (C), 147.3 (C), 133.2 (C), 131.7 (CH), 129.1 (CH), 128.7 (CH), 128.6 (CH), 128.4 (CH), 128.2 (CH),

126.7 (CH), 125.9 (CH), 122.5 (C), 117.5 (CH), 113.3 (CH), 100.1 (C), 97.4 (C), 67.5 (C). ^{77}Se-NMR (76 MHz, $CDCl_3$) δ (ppm): 119.5 (s). FTIR (KBr): 2149 (C≡N) cm^{-1}. HRMS: *m*/*z* $[M+H]^+$ calculated for $C_{21}H_{15}N_2Se$: 375.0400. found: 375.0411.

3-{[2-(4-Methoxyphenyl)ethynyl]selanyl}-2-phenylimidazo[1,2-*a*]pyridine (**4ab**)

Colorless needle (231 mg, 57%), m.p. 120–122°C (CH_2Cl_2/hexane). ^{1}H-NMR (400 MHz, $CDCl_3$) δ (ppm): 8.58 (dd, *J* = 6.9, 0.9 Hz, 1H), 8.13 (d, *J* = 7.3 Hz, 2H), 7.72 (d, *J* = 9.2 Hz, 1H), 7.52 (td, *J* = 7.3, 1.8 Hz, 2H), 7.43 (tt, *J* = 6.8, 1.3 Hz, 1H), 7.38–7.26 (m, 3H), 7.01 (t, *J* = 6.9 Hz, 1H), 6.78 (dt, *J* = 8.7, 1.8 Hz, 2H), 3.77 (s, 3H). ^{13}C-NMR (100 MHz, $CDCl_3$) δ (ppm): 159.9 (C), 150.1 (C), 147.3 (C), 133.5 (CH), 133.3 (C), 129.1 (CH), 128.5 (CH), 128.4 (CH), 126.6 (CH), 126.0 (CH), 117.5 (CH), 114.6 (C), 113.9 (CH), 113.2 (CH), 100.4 (C), 97.4 (C), 65.7 (C), 55.2 (CH_3). ^{77}Se-NMR (76 MHz, $CDCl_3$) δ (ppm): 117.4 (s). FTIR (KBr): 2160 (C≡N) cm^{-1}. HRMS: *m*/*z* $[M+H]^+$ calculated for $C_{22}H_{17}N_2OSe$: 405.0502. found: 405.0494.

3-{[2-(4-Methylphenyl)ethynyl]selanyl}-2-phenylimidazo[1,2-*a*]pyridine (**4ac**)

Colorless powder (194 mg, 51%), m.p. 100–101°C (CH_2Cl_2/hexane). ^{1}H-NMR (400 MHz, $CDCl_3$) δ (ppm): 8.59 (dd, *J* = 6.0, 0.9 Hz, 1H), 8.15 (d, *J* = 4.9 Hz, 2H), 7.75 (d, *J* = 8.9 Hz, 1H), 7.54 (t, *J* = 6.4 Hz, 2H), 7.44 (td, *J* = 6.4, 1.0 Hz, 1H), 7.37 (td, *J* = 6.8, 1.4 Hz, 1H), 7.27 (dd, *J* = 5.9, 2.3 Hz, 2H), 7.09–7.01 (m, 3H), 2.32 (s, 3H). ^{13}C-NMR (100 MHz, $CDCl_3$) δ (ppm): 150.1 (C), 147.3 (C), 139.0 (C), 133.2 (C), 131.7 (CH), 129.1 (CH), 129.0 (CH), 128.6 (CH), 128.4 (CH), 126.6 (CH), 126.0 (CH), 119.4 (C), 117.5 (CH), 113.2 (CH), 100.3 (C), 97.6 (C), 66.5 (C), 21.5 (CH_3). ^{77}Se-NMR (76 MHz, $CDCl_3$) δ (ppm): 118.3 (s). FTIR (KBr): 2155 (C≡N) cm^{-1}. HRMS: *m*/*z* $[M+H]^+$ calculated for $C_{22}H_{17}N_2Se$: 389.0553. found: 389.0547.

3-{[2-(4-Bromophenyl)ethynyl]selanyl}-2-phenylimidazo[1,2-*a*]pyridine (**4ad**)

Colorless plate (185 mg, 41%), m.p. 150–151°C (CH_2Cl_2/hexane). ^{1}H-NMR (400 MHz, $CDCl_3$) δ (ppm): 8.54 (d, *J* = 5.9 Hz, 1H), 8.12 (d, *J* = 7.8 Hz, 2H), 7.78–7.73 (m, 1H), 7.51 (t, *J* = 7.8 Hz, 2H), 7.45–7.37 (m, 4H), 7.19 (d, *J* = 7.8 Hz, 2H), 7.06–7.02 (m, 1H). ^{13}C-NMR (100 MHz, $CDCl_3$) δ (ppm): 150.5 (C), 147.4 (C), 133.3 (CH), 133.1 (C), 131.5 (CH), 129.1 (CH), 128.6 (CH), 128.4 (CH), 126.6 (CH), 125.8 (CH), 123.0 (C), 121.4 (C), 117.6 (CH), 113.5 (C), 113.3 (CH), 99.7 (C), 69.2 (C). ^{77}Se-NMR (76 MHz, $CDCl_3$) δ (ppm): 120.0 (s). FTIR (KBr): 2152 (C≡N) cm^{-1}. HRMS: *m*/*z* $[M+H]^+$ calculated for $C_{21}H_{14}BrN_2Se$: 452.9498. found: 452.9493.

3-{[2-(4-Trifluoromethylphenyl)ethynyl]selanyl}-2-phenylimidazo[1,2-*a*]pyridine (**4ae**)

Colorless needle (213 mg, 48%), m.p. 126–127°C (CH_2Cl_2/hexane). ^{1}H-NMR (400 MHz, $CDCl_3$) δ (ppm): 8.55 (d, *J* = 6.9 Hz, 1H), 8.12 (d, *J* = 8.2 Hz, 2H), 7.73 (d, *J* = 9.1 Hz, 1H), 7.54–7.50 (m, 4H), 7.45–7.42 (m, 3H), 7.37 (td, *J* = 6.9, 1.4 Hz, 1H), 7.03 (t, *J* = 6.9 Hz, 1H). ^{13}C-NMR (100 MHz, $CDCl_3$) δ (ppm): 150.8 (C), 147.6 (C), 133.3 (C), 131.8 (CH), 130.1 (q, *J* = 33 Hz, C), 129.1 (CH), 128.4 (CH), 126.6 (CH), 126.3 (C), 125.8 (CH), 125.2 (q, *J* = 3.9 Hz, CH), 123.9 (q, *J* = 273 Hz, C), 117.7 (CH), 113.3 (CH), 99.3 (C), 96.0 (C), 71.3 (C). ^{19}F-NMR (376 MHz, $CDCl_3$) δ (ppm): −64.1 (s). ^{77}Se-NMR (76 MHz, $CDCl_3$) δ (ppm): 121.4 (s). FTIR (KBr): 2554 (C≡N) cm^{-1}. HRMS: *m/z* $[M+H]^+$ calculated for $C_{22}H_{14}F_3N_2Se$: 443.0270. found: 443.0265.

2-Phenyl-3-{[2-(2-thienyl)ethynyl]selanyl}imidazo[1,2-*a*]pyridine (**4af**)

Yellow plate (232 mg, 61%), m.p. 78–80°C (AcOEt/Et_2O/hexane). ^{1}H-NMR (400 MHz, $CDCl_3$) δ (ppm): 8.55 (dt, *J* = 6.9 Hz, 1H), 8.13 (d, *J* = 7.3 Hz, 2H), 7.70 (d, *J* = 9.1 Hz, 1H), 7.51 (t, *J* = 6.0 Hz, 2H), 7.43 (d, *J* = 6.9 Hz, 1H), 7.40 (dd, *J* = 3.1, 1.0 Hz, 1H), 7.33 (ddd, *J* = 9.1, 6.9, 1.4 Hz, 1H), 7.19 (dd, *J* = 5.1, 3.1 Hz, 1H), 7.03 (dd, *J* = 5.0, 1.1 Hz, 1H), .6.99 (td, *J* = 6.7, 1.1 Hz, 1H). ^{13}C-NMR (100 MHz, $CDCl_3$) δ (ppm): 150.5 (C), 147.4 (C), 133.4 (C), 130.0 (CH), 129.8 (CH), 129.0 (CH), 128.5 (CH), 128.3 (CH), 126.4 (CH), 125.9 (CH), 125.3 (CH), 121.6 (C), 117.6 (CH), 113.1 (CH), 99.9 (C), 92.2 (C), 67.3 (C). ^{77}Se-NMR (76 MHz, $CDCl_3$) δ (ppm): 117.9 (s). FTIR (KBr): 2153 (C≡N) cm^{-1}. HRMS: *m/z* $[M+H]^+$ calculated for $C_{19}H_{13}N_2SSe$: 380.9959. found:.380.9965.

3-{[2-(1-Cyclohexenyl)ethynyl]selanyl}-2-phenylimidazo[1,2-*a*]pyridine (**4ag**)

Colorless needle (269 mg, 71%), m.p. 93–94°C (CH_2Cl_2/hexane). ^{1}H-NMR (400 MHz, $CDCl_3$) δ (ppm): 8.51 (dd, *J* = 5.9, 1.0 Hz, 1H), 8.10 (d, *J* = 8.2 Hz, 2H), 7.69 (dd, *J* = 8.2, 1.0 Hz, 1H), 7.50 (t, *J* = 7.8 Hz, 2H), 7.41 (td, *J* = 7.4, 0.9 Hz, 1H), 7.34 (t, *J* = 7.7 Hz, 1H), 6.99 (t, *J* = 6.8 Hz, 1H), 6.10–6.03 (m, 1H), 2.05–2.03 (m, 4H), 1.60–1.49 (m, 4H). ^{13}C-NMR (100 MHz, $CDCl_3$) δ (ppm): 150.2 (C), 147.4 (C), 136.7 (CH), 133.5 (C), 129.1 (CH), 128.4 (CH), 128.3 (CH), 126.3 (CH), 126.0 (CH), 120.4 (C), 117.5 (CH), 112.9 (CH), 100.5 (C), 99.3 (C), 64.1 (C), 28.9 (CH_2), 25.6 (CH_2), 22.1 (CH_2), 21.2 (CH_2). ^{77}Se-NMR (76 MHz, $CDCl_3$) δ (ppm): 115.9 (s). FTIR (KBr): 2147 (C≡N) cm^{-1}. HRMS: *m/z* $[M+H]^+$ calculated for $C_{21}H_{19}N_2Se$: 379.0700. found: 375.0411.

3-(1-Hexyn-1-ylselanyl)-2-phenylimidazo[1,2-*a*]pyridine (**4ah**)

Pale yellow oil (111 mg, 31%). ^{1}H-NMR (400 MHz, $CDCl_3$) δ (ppm): 8.50 (d, *J* = 6.9 Hz, 1H), 8.10 (dd, *J* = 7.4, 1.0 Hz, 2H), 7.70 (d, *J* = 9.1 Hz, 1H), 7.49 (t, *J* = 7.4 Hz, 2H), 7.41 (t, *J* = 6.9 Hz, 1H), 7.34 (t, *J* = 7.4 Hz, 1H), 6.98 (t, *J* = 6.9 Hz, 1H), 2.25 (t, *J* = 6.9 Hz, 2H), 1.43 (qui, *J* = 6.9 Hz, 2H), 1.32 (six, *J* = 6.9 Hz, 2H), 0.85 (t, *J* = 7.0 Hz, 3H). ^{13}C-NMR (100 MHz, $CDCl_3$) δ (ppm): 150.1 (C), 147.3 (C), 133.6 (C), 129.0 (CH), 128.4 (CH), 128.3 (CH), 126.2 (CH), 126.0 (CH), 117.5 (CH), 112.8 (CH), 100.8 (C), 98.9 (C), 56.2 (C), 30.4 (CH_2), 21.8 (CH_2), 19.8 (CH_2), 13.5 (CH_3). ^{77}Se-NMR (76 MHz, $CDCl_3$) δ (ppm): 111.2 (s). FTIR (neat): 2180 (C≡N) cm^{-1}. HRMS: *m*/*z* $[M+H]^+$ calculated for $C_{19}H_{19}N_2Se$: 355.0709. found: 355.0713.

6-Methoxy-2-phenyl-3-[(2-phenylethynyl)selanyl]imidazo[1,2-*a*]pyridine (**4ba**)

Colorless powder (279 mg, 69%), m.p. 118–120°C (CH_2Cl_2/hexane). ^{1}H-NMR (400 MHz, $CDCl_3$) δ (ppm): 8.12–8.08 (m, 3H), 7.65 (d, *J* = 9.6 Hz, 1H), 7.51 (t, *J* = 6.0 Hz, 2H), 7.48–7.32 (m, 3H), 7.31–7.25 (m, 3H), 7.16 (dd, *J* = 9.6, 2.3 Hz, 1H), 3.93 (s, 3H). ^{13}C-NMR (100 MHz, $CDCl_3$) δ (ppm): 149.9 (C), 144.0 (C), 133.2 (C), 131.9 (C), 131.7 (CH), 128.9 (CH), 128.7 (CH), 128.5 (CH), 128.4 (CH), 128.3 (CH), 122.5 (C), 121.7 (CH), 117.6 (CH), 107.8 (CH), 100.8 (C), 97.6 (C), 67.3 (C), 56.3 (CH_3). ^{77}Se-NMR (76 MHz, $CDCl_3$) δ (ppm): 122.8 (s). FTIR (KBr): 2155 (C≡N) cm^{-1}. HRMS: *m*/*z* $[M+H]^+$ calculated for $C_{22}H_{17}N_2OSe$: 405.0502. found: 405.0490.

6-Methyl-2-phenyl-3-[(2-phenylethynyl)selanyl]imidazo[1,2-*a*]pyridine (**4ca**)

Colorless needle (225 mg, 58%), m.p. 137–138°C (CH_2Cl_2/hexane). ^{1}H-NMR (400 MHz, $CDCl_3$) δ (ppm): 8.35 (s, 1H), 8.13 (d, *J* = 7.8 Hz, 2H), 7.64 (d, *J* = 9.1 Hz, 1H), 7.51 (t, *J* = 7.3 Hz, 2H), 7.48–7.20 (m, 7H), 2.44 (s, 3H). ^{13}C-NMR (100 MHz, $CDCl_3$) δ (ppm): 150.0 (C), 146.3 (C), 133.3 (C), 131.7 (CH), 129.8 (CH), 129.0 (CH), 128.6 (CH), 128.5 (CH), 128.4 (CH), 128.2 (CH), 123.7 (CH), 123.1 (C), 122.5 (C), 116.8 (CH), 99.6 (C), 97.2 (C), 68.0 (C), 18.5 (CH_3). ^{77}Se-NMR (76 MHz, $CDCl_3$) δ (ppm): 118.7 (s). FTIR (KBr): 2153 (C≡N) cm^{-1}. HRMS: *m*/*z* $[M+H]^+$ calculated for $C_{22}H_{17}N_2Se$: 389.0553. found: 389.0542.

6-Fluoro-2-phenyl-3-[(2-phenylethynyl)selanyl]imidazo[1,2-*a*]pyridine (**4da**)

Colorless needle (302 mg, 77%), m.p. 105–107°C (CH_2Cl_2/hexane). ^{1}H-NMR (400 MHz, $CDCl_3$) δ (ppm): 8.54–8.52 (m, 1H), 8.14–8.11 (m, 2H), 7.73 (dd, *J* = 9.6, 5.0 Hz, 1H), 7.53 (td, *J* = 7.8, 2.3 Hz, 2H), 7.45 (t, *J* = 5.0 Hz, 1H), 7.43–7.33 (m, 2H), 7.31–7.26 (m, 4H). ^{13}C-NMR (100 MHz, $CDCl_3$) δ (ppm): 153.7 (d, *J* = 239 Hz, C), 151.3 (C), 144.9 (C), 133.0 (C), 131.8 (C), 128.9 (CH), 128.84 (CH), 128.77 (CH), 128.5 (CH), 128.3 (CH), 122.3 (CH),

118.5 (d, J = 25 Hz, CH), 118.0 (d, J = 8.7 Hz, CH), 112.9 (d, J = 41 Hz, CH), 101.6 (C), 97.9 (C), 66.8 (C). ^{19}F-NMR (376 MHz, $CDCl_3$) δ (ppm): −139.0 (s). ^{77}Se-NMR (76 MHz, $CDCl_3$) δ (ppm): 124.1 (s). FTIR (KBr): 2154 (C≡N) cm^{-1}. HRMS: m/z $[M+H]^+$ calculated for $C_{21}H_{14}FN_2Se$: 393.0302. found: 393.0292.

6-Bromo-2-phenyl-3-[(2-phenylethynyl)selanyl]imidazo[1,2-*a*]pyridine (**4ea**)

Colorless needle (187 mg, 41%), m.p. 147–149°C (CH_2Cl_2/hexane). ^{1}H-NMR (400 MHz, $CDCl_3$) δ (ppm): 8.72 (s, 1H), 8.11 (d, J = 6.9 Hz, 2H), 7.65 (d, J = 9.1 Hz, 1H), 7.53 (td, J = 8.2, 1.8 Hz, 2H), 7.47–7.38 (m, 4H), 7.33–7.26 (m, 3H). ^{13}C-NMR (100 MHz, $CDCl_3$) δ (ppm): 150.5 (C), 145.6 (C), 132.6 (C), 131.9 (CH), 130.3 (CH), 129.0 (CH), 128.94 (CH), 128.91 (CH), 128.5 (CH), 128.3 (CH), 126.2 (CH), 122.2 (C), 118.1 (CH), 108.2 (C), 100.7 (C), 98.1 (C), 66.7 (C). ^{77}Se-NMR (76 MHz, $CDCl_3$) δ (ppm): 124.4 (s). FTIR (KBr): 2156 (C≡N) cm^{-1}. HRMS: m/z $[M+H]^+$ calculated for $C_{21}H_{14}BrN_2Se$: 452.9498. found: 452.9488.

2-(4-Methoxyphenyl)-3-[(2-phenylethynyl)selanyl]imidazo[1,2-*a*]pyridine (**4ga**)

Colorless needle (274 mg, 68%), m.p. 147–149°C (CH_2Cl_2/hexane). ^{1}H-NMR (400 MHz, benzene-d_6) δ (ppm): 8.57 (d, J = 8.5 Hz, 2H), 8.12 (d, J = 6.9 Hz, 1H), 7.44 (d, J = 8.7 Hz, 1H), 7.20 (dd, J = 7.3, 0.9 Hz, 2H), 6.94 (d, J = 8.5 Hz, 2H), 6.87–6.79 (m, 3H), 6.59 (t, J = 7.8 Hz, 1H), 6.19 (t, J = 6.8 Hz, 1H), 3.30 (s, 3H). ^{13}C-NMR (100 MHz, benzene-d_6) δ (ppm): 160.6 (C), 151.2 (C), 147.8 (C), 132.0 (CH), 130.9 (CH), 128.6 (CH), 128.5 (CH), 128.3 (CH), 127.2 (C), 125.7 (CH), 123.2 (C), 117.8 (CH), 114.3 (CH), 112.5 (CH), 99.0 (C), 97.4 (C), 69.5 (C), 54.8 (CH_3). ^{77}Se-NMR (76 MHz, benzene-d_6) δ (ppm): 114.5 (s). FTIR (KBr): 2155 (C≡N) cm^{-1}. HRMS: m/z $[M+H]^+$ calculated for $C_{22}H_{17}N_2OSe$: 405.0502. found: 405.0495.

2-(4-Methylphenyl)-3-[(2-phenylethynyl)selanyl]imidazo[1,2-*a*]pyridine (**4ha**)

Colorless needle (236 mg, 61%), m.p. 111–113°C (CH_2Cl_2/hexane). ^{1}H-NMR (400 MHz, $CDCl_3$) δ (ppm): 8.57 (dd, J = 6.4, 0.9 Hz, 1H), 8.04 (dd, J = 6.4, 1.8 Hz, 2H), 7.75 (d, J = 9.2 Hz, 1H), 7.39–7.30 (m, 5H), 7.29–7.23 (m, 3H), 7.03 (t, J = 6.9 Hz, 1H), 2.43 (s, 3 H). ^{13}C-NMR (100 MHz, $CDCl_3$) δ (ppm): 150.3 (C), 147.3 (C), 138.8 (C), 131.9 (CH), 130.3 (C), 129.3 (CH), 129.1 (CH), 128.8 (CH), 128.4 (CH), 126.9 (CH), 126.1 (CH), 122.6 (C), 117.5 (CH), 113.4 (CH), 100.0 (C), 97.5 (C), 67.7 (C), 21.5 (CH_3). ^{77}Se-NMR (76 MHz, $CDCl_3$) δ (ppm): 119.0 (s). FTIR (KBr): 2153 (C≡N) cm^{-1}. HRMS: m/z $[M+H]^+$ calculated for $C_{22}H_{17}N_2Se$: 389.0553. found: 389.0553.

2-(4-Fluorophenyl)-3-[(2-phenylethynyl)selanyl]imidazo[1,2-*a*]pyridine (**4ia**)

Colorless needle (270 mg, 69%), m.p. 126–128°C (CH_2Cl_2/hexane). ^{1}H-NMR (400 MHz, $CDCl_3$) δ (ppm): 8.57 (d, *J* = 6.9 Hz, 1H), 8.15–8.11 (m, 2H), 7.72 (d, *J* = 8.7 Hz, 1H), 7.40–7.18 (m, 8H), 7.04 (t, *J* = 6.9 Hz, 1H). ^{13}C-NMR (100 MHz, $CDCl_3$) δ (ppm): 163.1 (d, *J* = 248 Hz, C), 149.4 (C), 147.3 (C), 131.7 (CH), 130.8 (d, *J* = 7.7 Hz, CH), 129.4 (C), 128.7 (CH), 128.3 (CH), 126.8 (CH), 125.9 (CH), 122.4 (C), 117.5 (CH), 115.4 (d, *J* = 21 Hz, CH), 113.4 (CH), 99.9 (C), 97.4 (C), 67.4 (C). ^{19}F-NMR (376 MHz, $CDCl_3$) δ (ppm): −114.2 (s). ^{77}Se-NMR (76 MHz, $CDCl_3$) δ (ppm): 117.6 (s). FTIR (KBr): 2156 (C≡N) cm^{-1}. HRMS: *m/z* $[M+H]^+$ calculated for $C_{21}H_{14}FN_2Se$: 393.0302. found: 393.0296.

2-(4-Chlorophenyl)-3-[(2-phenylethynyl)selanyl]imidazo[1,2-*a*]pyridine (**4ja**)

Colorless needle (270 mg, 66%), m.p. 134–137°C (CH_2Cl_2/hexane). ^{1}H-NMR (400 MHz, $CDCl_3$) δ (ppm): 8.57 (d, *J* = 6.9 Hz, 1H), 8.10 (d, *J* = 8.2 Hz, 2H), 7.73 (d, *J* = 8.7 Hz, 1H), 7.48 (d, *J* = 8.2 Hz, 2H), 7.49–7.34 (m, 3H), 7.31–7.24 (m, 3H), 7.04 (t, *J* = 6.9 Hz, 1H). ^{13}C-NMR (100 MHz, $CDCl_3$) δ (ppm): 149.1 (C), 147.3 (C), 134.6 (C), 131.7 (CH), 130.3 (CH), 128.8 (CH), 128.6 (CH), 128.3 (CH), 126.9 (CH), 125.9 (CH), 122.3 (C), 117.5 (CH), 113.4 (CH), 100.2 (C), 97.5 (C), 67.3 (C). ^{77}Se-NMR (76 MHz, $CDCl_3$) δ (ppm): 118.2 (s). FTIR (KBr): 2156 (C≡N) cm^{-1}. HRMS: *m/z* $[M+H]^+$ calculated for $C_{21}H_{14}ClN_2Se$: 409.0004. found: 408.9995.

2-(4-Bromophenyl)-3-[(2-phenylethynyl)selanyl]imidazo[1,2-*a*]pyridine (**4ka**)

Colorless needle (325 mg, 72%), m.p. 144–145°C (CH_2Cl_2/hexane). ^{1}H-NMR (400 MHz, $CDCl_3$) δ (ppm): 8.58 (d, *J* = 6.4 Hz, 1H), 8.04 (dt, *J* = 6.9, 2.3 Hz, 2H), 7.76 (t, *J* = 9.1 Hz, 1H), 7.65 (d, *J* = 8.2 Hz, 2H), 7.42–7.24 (m, 6H), 7.06 (t, *J* = 6.9 Hz, 1H). ^{13}C-NMR (100 MHz, $CDCl_3$) δ (ppm): 147.2 (C), 131.9 (C), 131.8 (CH), 131.6 (CH), 131.0 (CH), 130.6 (C), 128.8 (CH), 128.3 (CH), 127.1 (CH), 126.0 (CH), 123.1 (C), 122.3 (C), 117.4 (CH), 113.6 (CH), 100.4 (C), 97.6 (C), 67.1 (C). ^{77}Se-NMR (76 MHz, $CDCl_3$) δ (ppm): 118.6 (s). FTIR (KBr): 2155 (C≡N) cm^{-1}. HRMS: *m/z* $[M+H]^+$ calculated for $C_{21}H_{14}BrN_2Se$: 452.9498. found: 452.9487.

2-(4-Trifluoromethylphenyl)-3-[(2-phenylethynyl)selanyl]imidazo[1,2-*a*]pyridine (**4la**)

Colorless needle (225 mg, 51%), m.p. 123–124°C (CH_2Cl_2/hexane). ^{1}H-NMR (400 MHz, $CDCl_3$) δ (ppm): 8.61 (d, *J* = 6.9 Hz, 1H), 8.29 (d, *J* = 8.2 Hz, 2H), 7.79–7.76 (m, 3H), 7.42 (t, *J* = 8.0 Hz, 1H), 7.36 (dd, *J* = 7.4, 1.4 Hz, 2H), 7.31–7.25 (m, 3H), 7.08 (t, *J* = 6.9 Hz, 1H). ^{13}C-NMR (100 MHz, $CDCl_3$) δ (ppm): 148.5 (C), 147.4 (C), 136.7 (C), 131.8 (CH), 130.4 (q, *J* = 34 Hz, C), 129.3 (CH), 128.9 (CH), 128.3 (CH), 127.2 (CH), 126.0 (CH), 125.3 (q, *J*

= 3.9 Hz, CH), 124.2 (q, J = 273 Hz, C), 122.3 (C), 117.7 (CH), 113.7 (CH), 101.1 (C), 97.7 (C), 67.0 (C). ^{19}F-NMR (376 MHz, $CDCl_3$) δ (ppm): −63.9 (s). ^{77}Se-NMR (76 MHz, $CDCl_3$) δ (ppm): 118.6 (s). FTIR (KBr): 2158 (C≡N) cm^{-1}. HRMS: *m/z* $[M+H]^+$ calculated for $C_{22}H_{14}F_3N_2Se$: 443.0270. found: 443.0257.

2-(2-Methylphenyl)-3-[(2-phenylethynyl)selanyl]imidazo[1,2-*a*]pyridine (**4ma**)

Colorless needle (229 mg, 59%), m.p. 105–107°C (CH_2Cl_2/hexane). ^{1}H-NMR (400 MHz, $CDCl_3$) δ (ppm): 8.55 (d, J = 6.9 Hz, 1H), 7.73 (d, J = 8.7 Hz, 1H), 7.47 (t, J = 7.3 Hz, 1H), 7.40–7.26 (m, 9H), 7.04 (t, J = 6.8 Hz, 1H), 2.41 (s, 3H). ^{13}C-NMR (100 MHz, $CDCl_3$) δ (ppm): 152.0 (C), 147.2 (C), 137.6 (C), 133.0 (C), 131.7 (CH), 131.1 (CH), 130.3 (CH), 128.6 (CH), 128.3 (CH), 126.3 (CH), 125.9 (CH), 125.3 (CH), 122.6 (C), 117.6 (CH), 113.2 (CH), 101.9 (C), 97.3 (C), 67.7 (C), 20.4 (CH_3). ^{77}Se-NMR (76 MHz, $CDCl_3$) δ (ppm): 111.5 (s). FTIR (KBr): 2152 (C≡N) cm^{-1}. HRMS: *m/z* $[M+H]^+$ calculated for $C_{22}H_{17}N_2Se$: 389.0553. found: 389.0545.

Reaction of 3-(ethynylselanyl)imidazo[1,2-*a*]pyridine:

Reaction with phenyllithium

A solution of PhLi (1.12 M solution in cyclohexane/diethyl ether, 0.9 mL, 1.0 mmol, 2 equiv) was added dropwise to a solution of **4aa** (187 mg, 0.5 mmol) in dry THF (2 mL) at −78 °C under an Ar atmosphere. After 1 h, the reaction mixture was diluted with CH_2Cl_2 (20 mL) and water (20 mL) at 0 °C. The phases were separated, and the aqueous layer was extracted with CH_2Cl_2 (10 mL × 2). The combined organic layer was washed with water (10 mL × 3), dried over anhydrous magnesium sulfate, filtered, and concentrated under reduced pressure. The residue was purified by column chromatography using hexane/AcOEt as eluent to give 2-phenyl-3-(phenylselanyl)imidazo[1,2-*a*]pyridine (**6a**) [1] as a yellow oil (85 mg, 49%). ^{1}H-NMR (400 MHz, $CDCl_3$) δ (ppm): 8.36 (dt, J = 6.9, 1.4 Hz, 1H), 8.16 (d, J = 7.3 Hz, 2H), 7.78 (d, J = 9.1 Hz, 1H), 7.45 (tt, J = 6.9, 2.3 Hz, 2H), 7.41–7.33 (m, 2H), 7.20–7.16 (m, 3H), 7.13–7.10 (m, 2H), 6.89 (td, J = 6.9, 0.9 Hz, 1H). LRMS (EI) *m/z*: 350.0 ($[M]^+$, 40%), 270.1 (100%). HRMS: *m/z* $[M]^+$ calculated for $C_{19}H_{14}N_2Se$: 350.0322. Found: 350.0319.

Reaction with *n*-butyllithium

A solution of *n*-BuLi (1.55 M solution in hexane, 2.0 mL, 3.0 mmol, 6 equiv) was added dropwise to a solution of **4aa** (187 mg, 0.5 mmol) in dry THF (2 mL) at −78 °C under an Ar atmosphere. After 1 h, the reaction mixture was diluted with CH_2Cl_2 (20 mL) and water (20 mL) at 0 °C. The phases were separated, and the aqueous layer was

extracted with CH_2Cl_2 (10 mL × 2). The combined organic layer was washed with water (10 mL × 3), dried over anhydrous magnesium sulfate, filtered, and concentrated under reduced pressure. The residue was purified by column chromatography using hexane/AcOEt as eluent to give 3-(butylselanyl)-2-phenylimidazo[1,2-*a*]pyridine (**6b**) [2] as a yellow oil (104 mg, 63%). ^{1}H-NMR (400 MHz, $CDCl_3$) δ (ppm): 8.56 (d, *J* = 6.9 Hz, 1H), 8.24 (dt, *J* = 8.2, 1.8 Hz, 2H)), 7.69 (d, *J* = 9.1 Hz, 1H), 7.47 (td, *J* = 7.3, 1.4 Hz, 2H), 7.38 (tt, *J* = 7.8, 1.8 Hz, 1H), 7.29 (t, *J* = 7.8 Hz, 1H), 6.92 (t, *J* = 6.9 Hz, 1H), 2.68 (t, *J* = 7.8 Hz, 2H), 1.47 (qui, *J* = 7.3 Hz, 2H), 1.29 (six, *J* = 7.8 Hz, 2H), 0.76 (t, *J* = 7.3 Hz, 3H). LRMS (EI) *m/z*: 330.1 ($[M]^+$, 30%), 273.0 (100%) 194.1 (99%). HRMS: *m/z* $[M]^+$ calculated for $C_{17}H_{18}N_2Se$: 330.0635. Found: 330.0639.

Reaction with $TMSCF_3$

To a solution of **4aa** (187 mg, 0.5 mmol) and cesium carbonate (652 mg, 2.0 mmol, 4 equiv) in dry acetonitrile (4 mL) was added (trifluoromethyl)trimethylsilane (111 μL, 0.75 mmol, 1.5 equiv) at 0 °C. The solution was stirred for 1 h at 0 °C and then warmed to room temperature. After 48 h, the reaction mixture was filtered through a short pad of silica, rinsed with dichloromethane (30 mL) and the collected solution was concentrated under reduced pressure. The residue was purified by column chromatography using hexane/AcOEt as eluent to give 3-(trifluoromethylselanyl)-2-phenylimidazo[1,2-*a*]pyridine (**7**) [3] as a yellow oil (75 mg, 44%). ^{1}H-NMR (400 MHz, $CDCl_3$) δ (ppm): 8.54 (d, *J* = 6.9 Hz, 1H), 8.07 (d, *J* = 6.9 Hz, 2H), 7.73 (d, *J* = 8.8 Hz, 1H), 7.53–7.39 (m, 4H), 7.02 (td, *J* = 6.8, 1.0 Hz, 1H). LRMS (EI) *m/z*: 342.0 ($[M]^+$, 30%), 273.0 (100%), 78.0 (30%). HRMS: *m/z* $[M]^+$ calculated for $C_{14}H_9F_3N_2Se$: 341.9883. Found: 341.9882.

Reaction with benzylazide

A solution of **4aa** (187 mg, 0.5 mmol), benzylazide (147 mg, 1.1 mmol, 2.2 equiv) CuI (95 mg, 0.5 mmol, 1 equiv) and *N*,*N*,*N*',*N*'',*N*''-pentamethyldiethylenetriamine (87 mg, 0.5 mmol, 1 equiv) in dry THF (2 mL) was heated at 60 °C under an Ar atmosphere. After 5.5 h, the mixture was allowed to cool to room temperature, and diluted with AcOEt (20 mL) and saturated aqueous NH_4Cl (20 mL) at 0°C. The phases were separated and the aqueous layer was extracted with AcOEt (10 mL × 2). The combined organic layer was washed with saturated aqueous NH_4Cl (20 mL × 6), dried over anhydrous magnesium sulfate, filtered, and concentrated under reduced pressure. The residue was purified by column chromatography using hexane/AcOEt as eluent to give 3-[(1-benzyl-4-phenyl)-1*H*-1,2,3-triazolyl]-2-phenylimidazo[1,2-*a*]pyridine (**8**) as colorless prisms (182 mg, 72%). m.p. 118–121°C (EtOH). ^{1}H-NMR

(400 MHz, $CDCl_3$) δ (ppm): 7.92 (dd, *J* = 7.8, 1.8 Hz, 2H), 7.87–7.85 (m, 2H), 7.58–7.48 (m, 8H), 7.24–7.14 (m, 4H), 6.64 (d, *J* = 6.9 Hz, 2H), 6.44 (td, *J* = 6.9, 0.9 Hz, 1H), 5.11 (s, 2H). ^{13}C-NMR (100 MHz, $CDCl_3$) δ (ppm): 151.7 (C), 151.2 (C), 147.0 (C), 134.6 (C), 132.9 (C), 130.5 (C), 129.7 (CH), 129.1 (CH), 128.9 (CH), 128.7 (CH), 128.62 (CH), 128.57 (CH), 128.5 (CH), 128.0 (CH), 126.9 (CH), 126.6 (CH), 125.5 (CH), 117.6 (C), 117.3 (CH), 113.1 (CH), 102.1 (C), 52.6 (CH_2). ^{77}Se-NMR (76 MHz, $CDCl_3$) δ (ppm): 46.8 (s). IR (KBr): *v* = 3422, 3065, 3028, 1460, 1343 cm^{-1}. HRMS: *m*/*z* $[M]^+$ calculated for $C_{28}H_{21}N_5Se$: 507.0962. found: 507.0966.

3. Single crystal X-ray diffraction experiment

A suitable crystal was selected and measured on an XtaLAB Synergy, Single source at home/near, HyPix3000 diffractometer (Rigaku, Tokyo, Japan). The crystal was kept at 103 K in the N_2 cold stream during data collection. Using Olex2 [4], the structure was solved with the SHELXT [5] structure solution program using Intrinsic Phasing and refined with the SHELXL [6] refinement package using Least Squares minimization. All non-hydrogen atoms were refined anisotropically. The hydrogen atoms were refined isotropically on the calculated positions using a riding model (AFIX 23 and 43) with U_{iso} values constrained to 1.2/1.5 U_{eq} of their parent atoms.

Data of compound **4aa**

The colorless prism crystal (0.105 × 0.096 × 0.056 mm^3), obtained from CH_2Cl_2/hexane, was immersed in Paraton-N oil. $C_{21}H_{14}N_2Se$, Mr = 373.30; monoclinic, space group $P2_1/c$, $Z = 4$, D_{calc} = 1.506 g·cm^{-3}, μ(Cu Kα) = 3.097 mm^{-1}, a = 8.60000(10), b = 11.4839(2), c = 16.6687(2) Å, β = 90.8590(10)°, V = 1646.04(4) Å^3, 7991 measured and 2989 independent [$I > 2\sigma(I)$] reflections, 217 parameters, final R_1 = 0.0274, wR_2 = 0.0667, S = 1.056 [$I > 2\sigma(I)$]. CCDC 2156343.

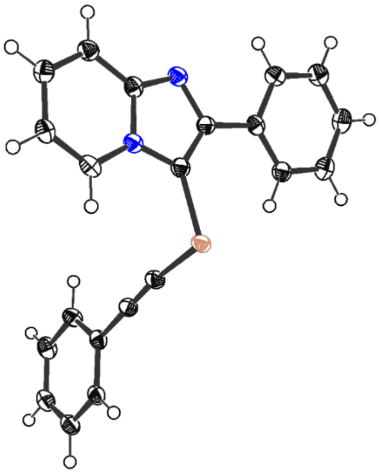

Figure S1. Ortep drawing of **2a** (50% probability)

Data of compound **8**

The colorless prism crystal (0.296 × 0.195 × 0.064 mm^3), obtained from EtOH, was immersed in Paraton-N oil. $C_{28}H_{21}N_5Se$, Mr = 506.46; triclinic, space group P−1, Z = 2, D_{calc} = 1.473 g·cm^{-3}, μ(Cu Kα) = 2.434 mm^{-1}, a = 9.1538(2), b = 10.0410(3), c = 13.2207(6) Å, α = 105.832(3), β = 90.8590(10), γ = 100.780(2)°, V = 1141.77(7) Å^3, 9609 measured and 9609 independent [$I > 2\sigma(I)$] reflections, 308 parameters, final R_1 = 0.0459, wR_2 = 0.1285, S = 1.084 [$I > 2\sigma(I)$]. CCDC 2156344.

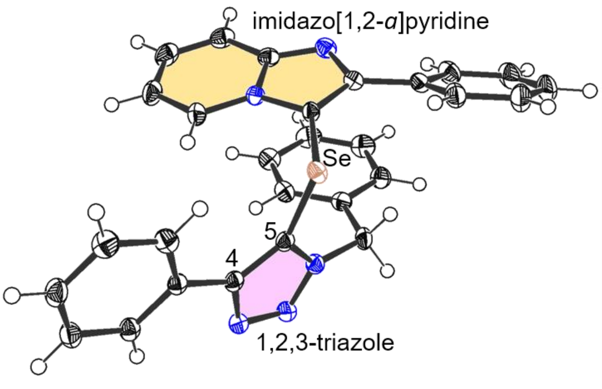

Figure S2. Ortep drawing of 8 (50% probability)

4. References

1. Kondo, K.; Matsumura, M.; Kanasaki, K.; Murata, Y.; Kakusawa, N.; Yasuike, S. *Synthesis* **2018**, *50*, 2200–2210.
2. Vieira, B. M.; Thurow, S.; da Costa, M.; Casaril, A. M.; Domingues, M.; Schumacher, R. F.; Perin, G.; Alves, D.; Savegnago, L.; Lenardão, E. J. *Asian J. Org. Chem*. **2017**, *6*, 1635–1646.
3. Redon, S.; Obah Kosso, A. R.; Broggi, J.; Vanelle, P. *Tetrahedron Lett.* **2017**, *58*, 2771–2773.
4. Dolomanov, O. V.; Bourhis, L. J.; Gildea, R. J.; Howard, J. A. K.; Puschmann, H. *J. Appl. Cryst.* **2009**, *42*, 339–341.
5. Sheldrick, G. M. *Acta Cryst.* **2015**, *A71*, 3–8.
6. Sheldrick, G. M. *Acta Cryst.* **2015**, *C71*, 3–8.

NMR data of novel compounds

^{1}H NMR of **4aa**

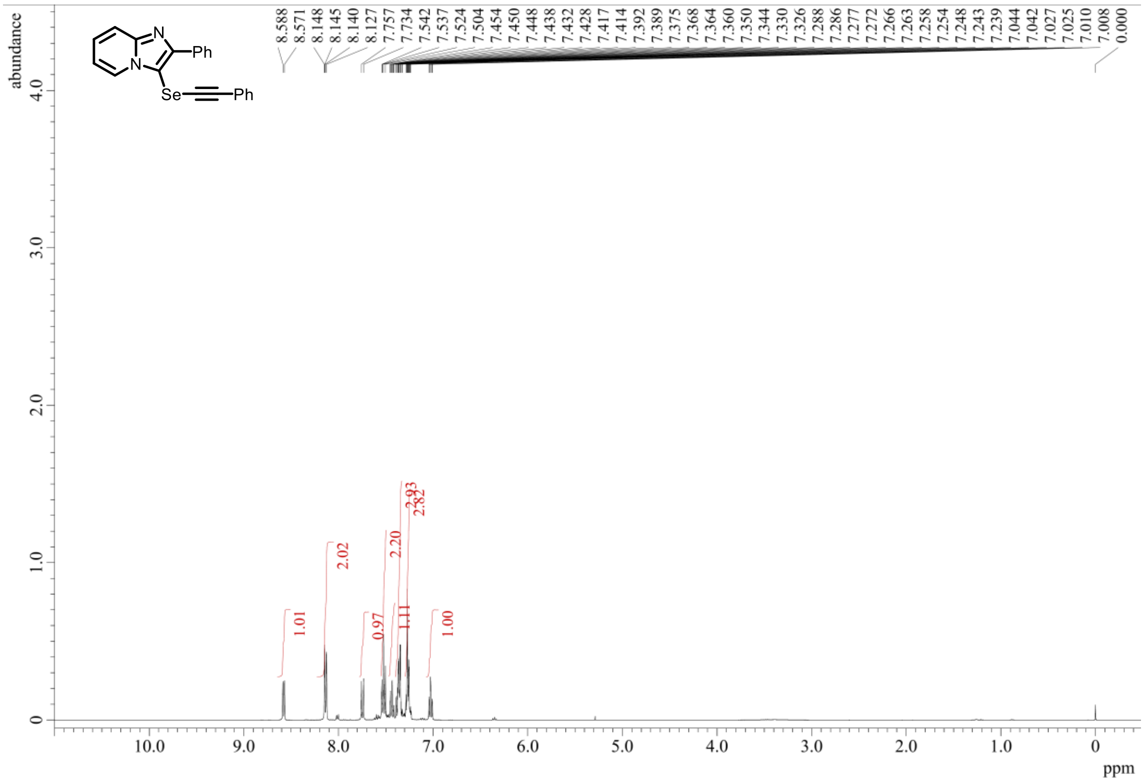

^{13}C NMR of **4aa**

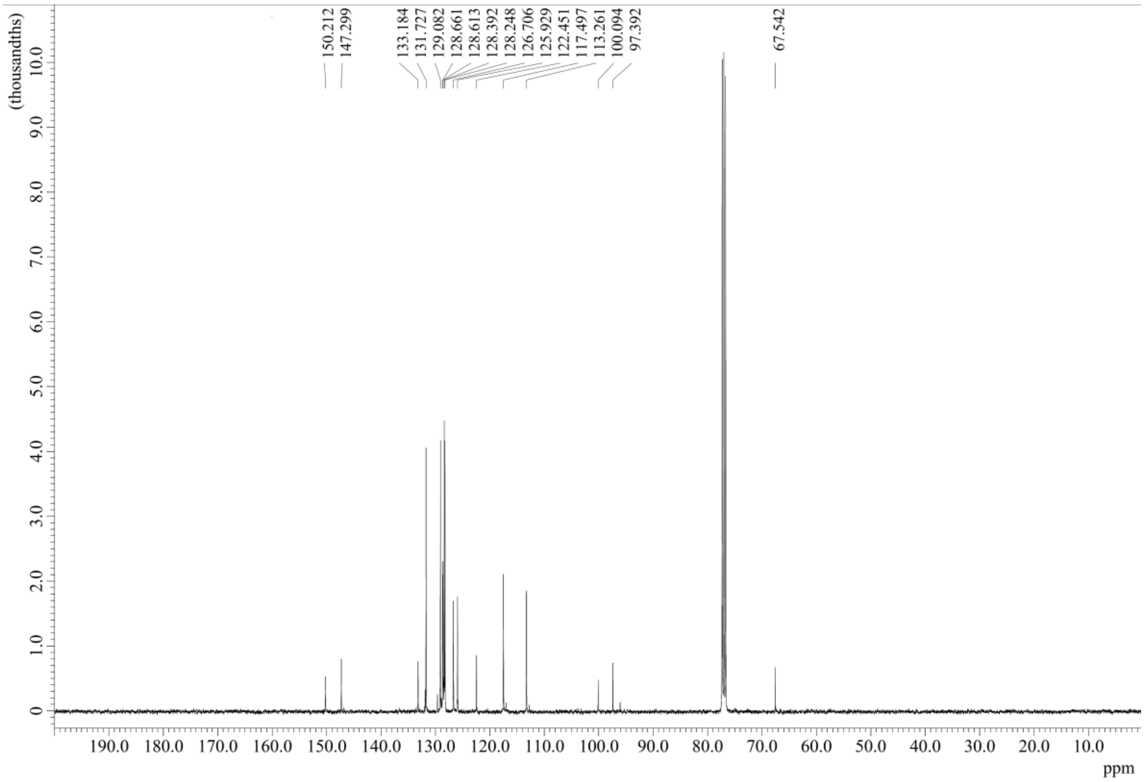

^{1}H NMR of **4ab**

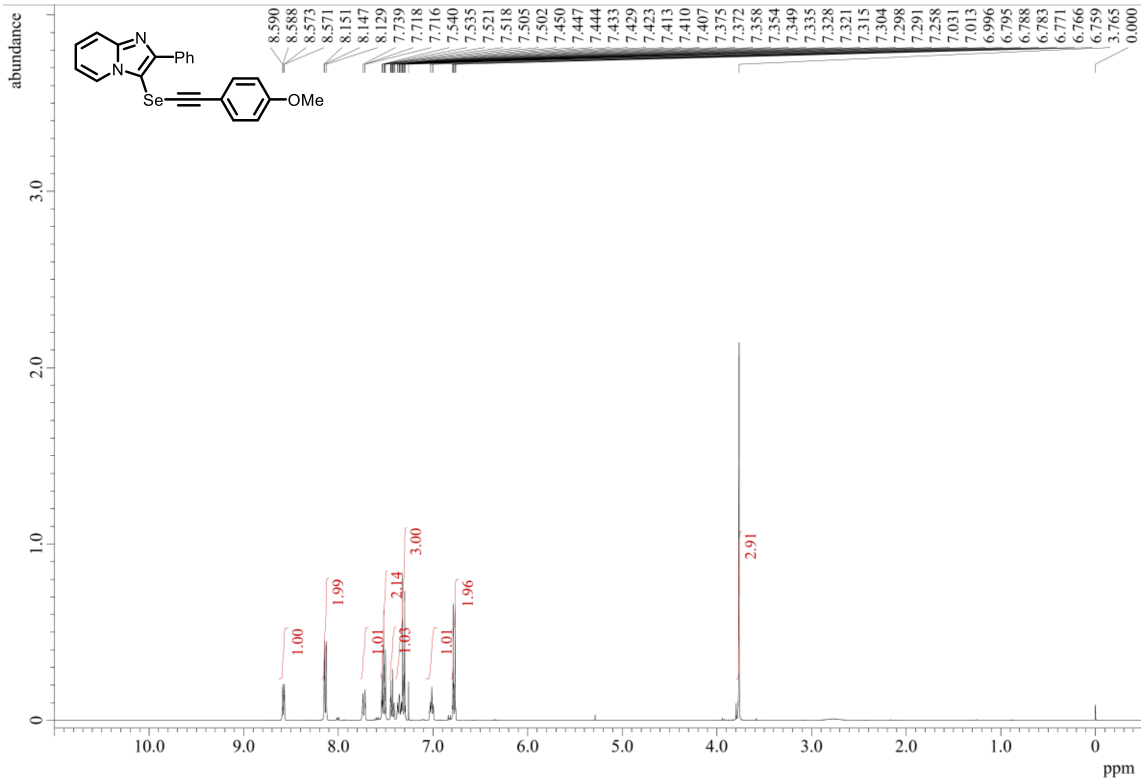

^{13}C NMR of **4ab**

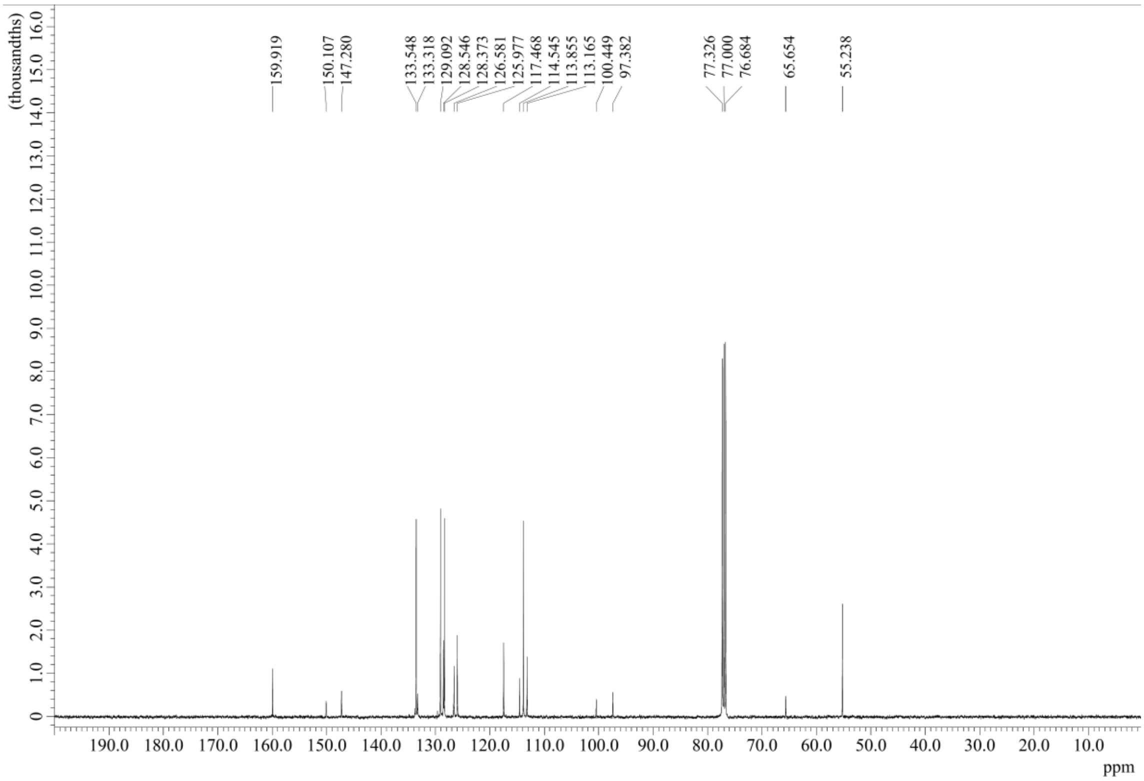

^{1}H NMR of **4ac**

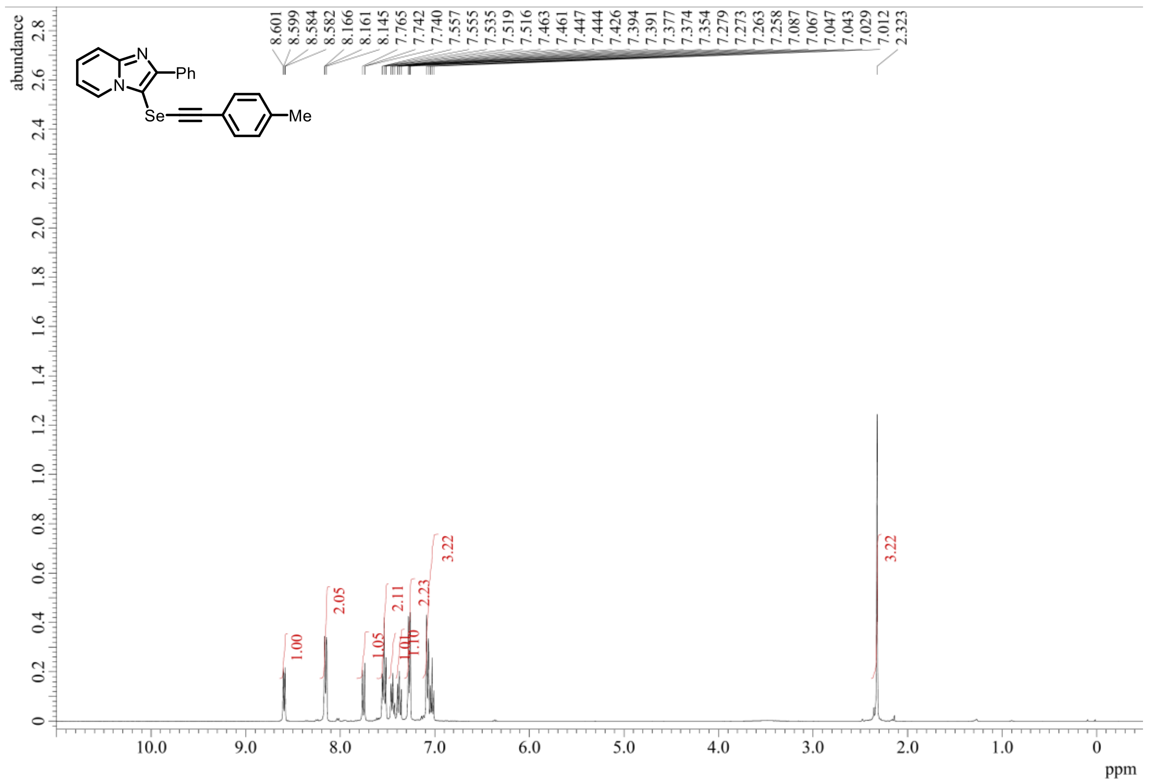

^{13}C NMR of **4ac**

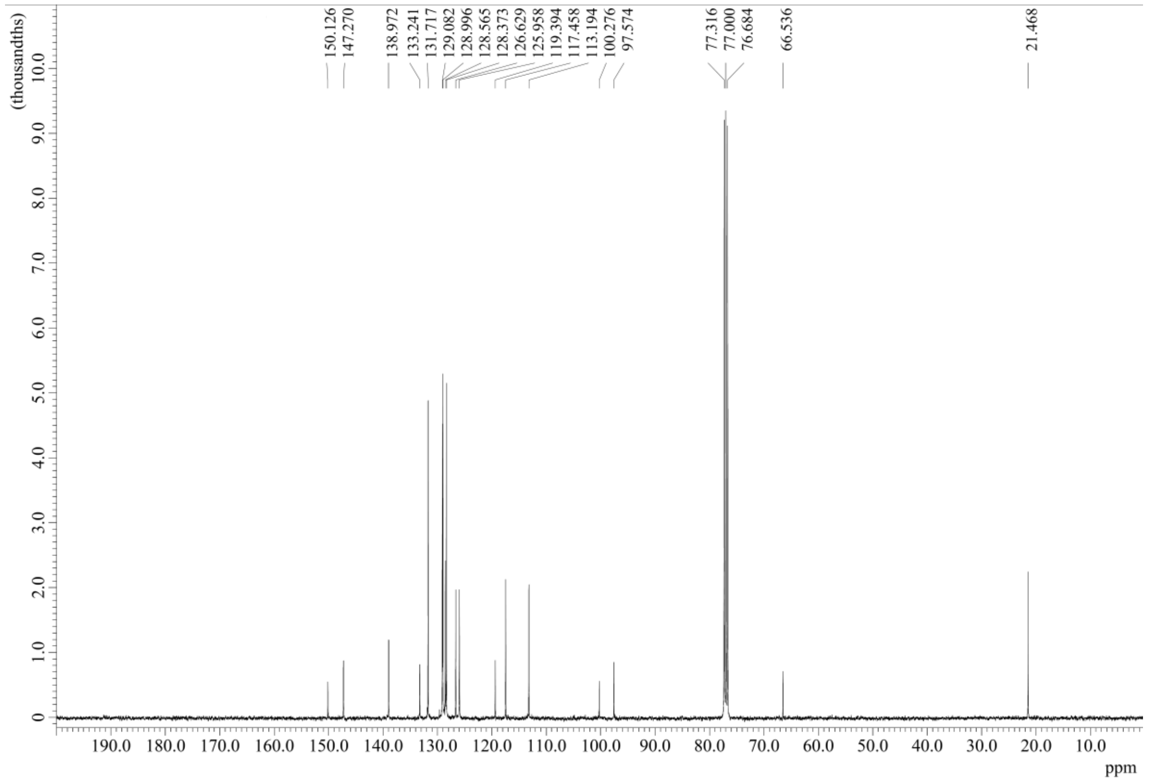

^{1}H NMR of **4ad**

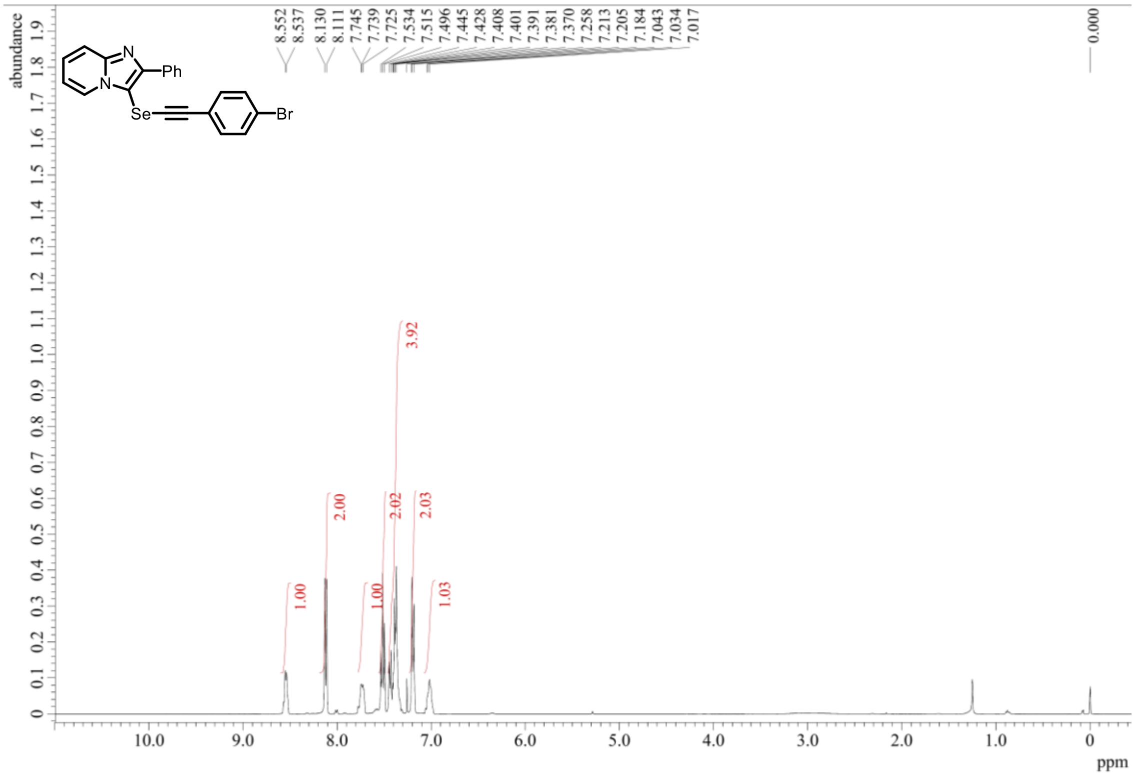

^{13}C NMR of **4ad**

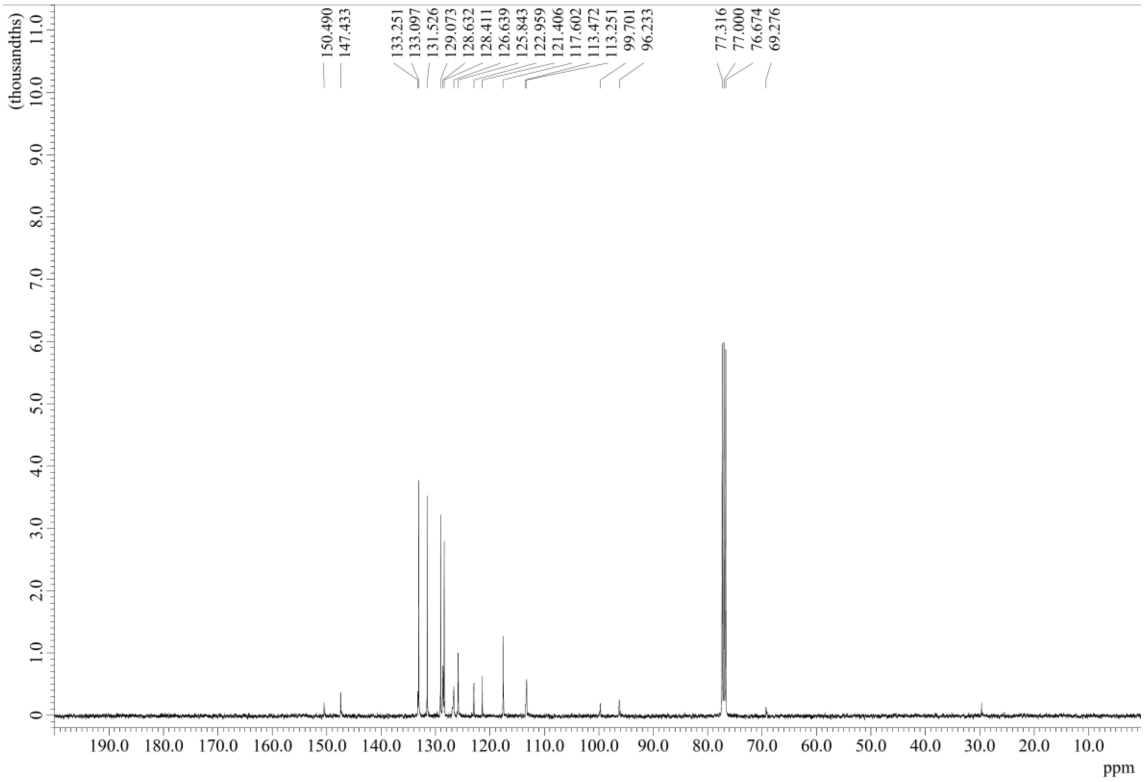

^{1}H NMR of **4ae**

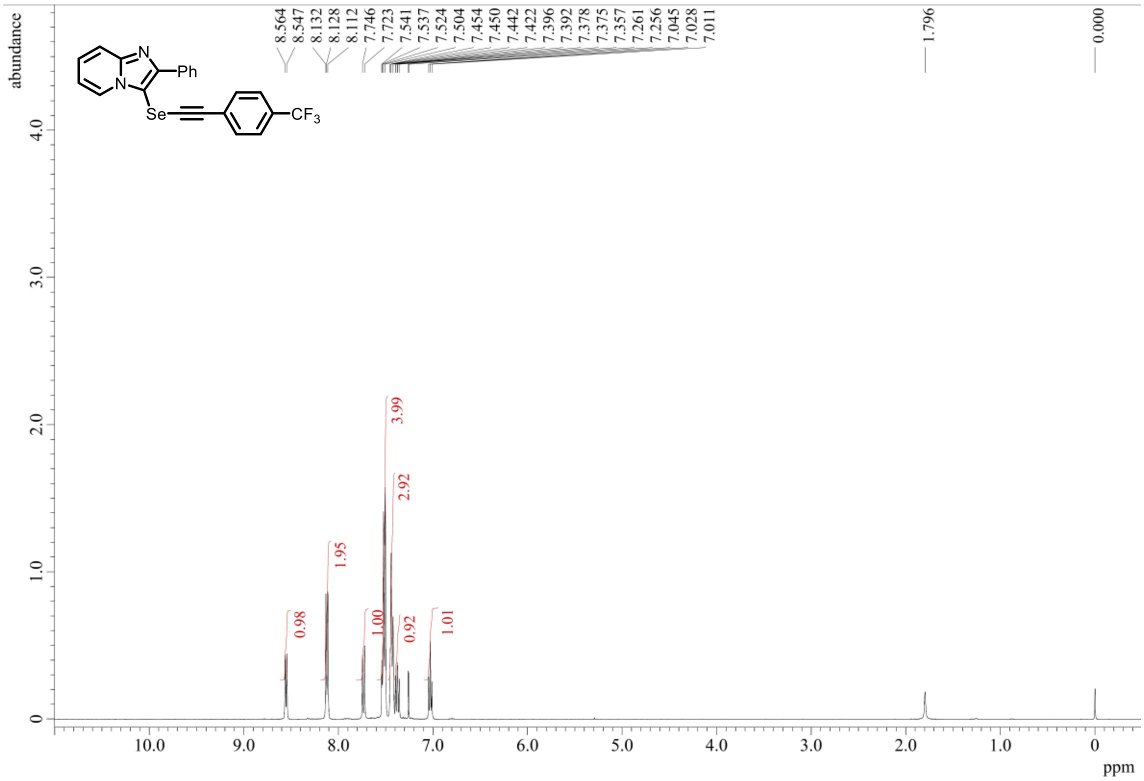

^{13}C NMR of **4ea**

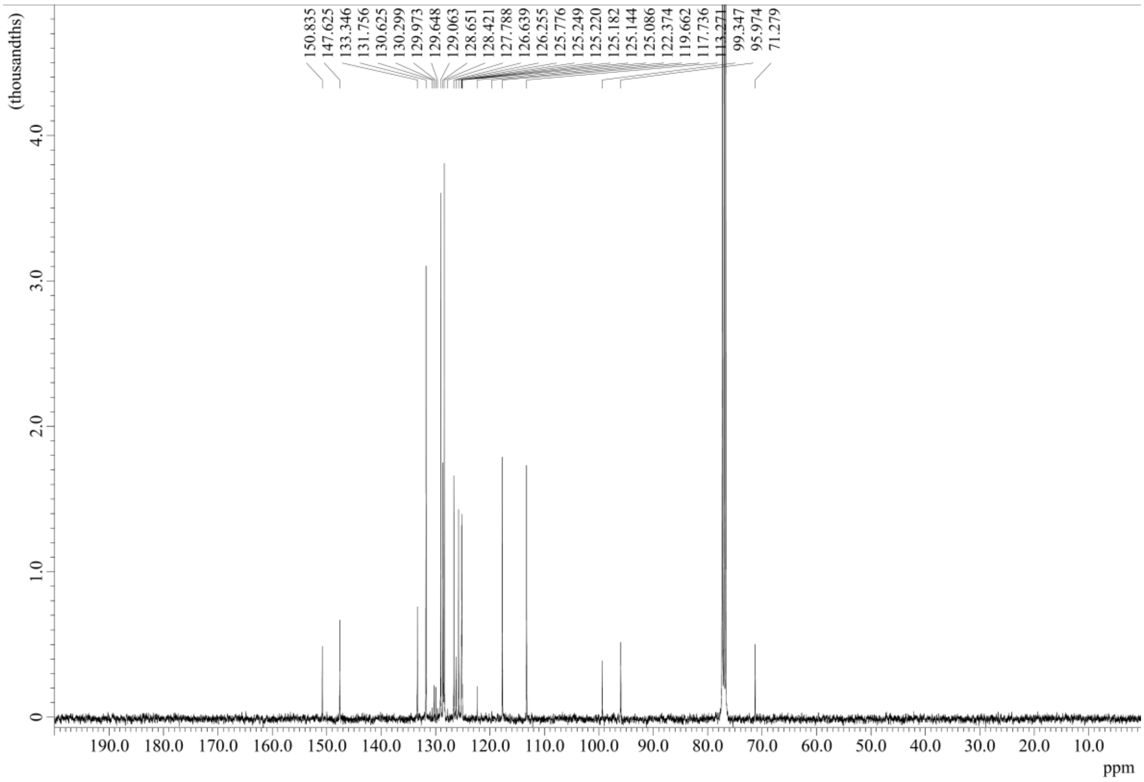

^{1}H NMR of **4af**

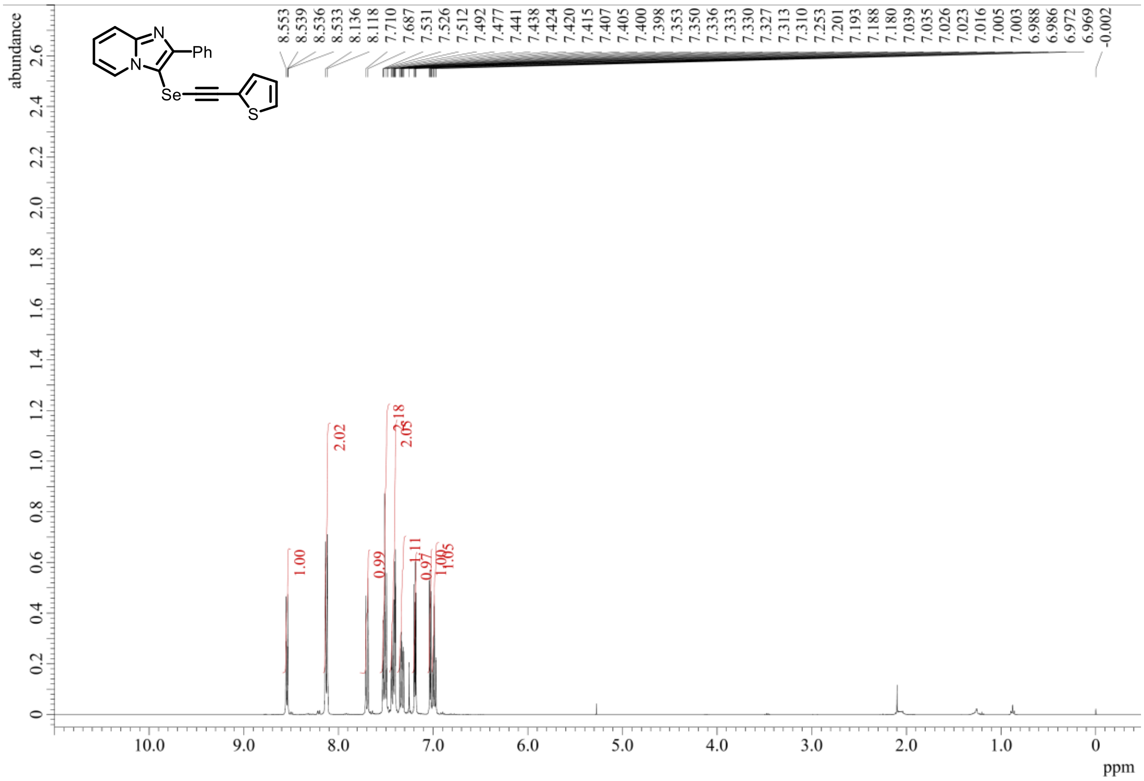

^{13}C NMR of **4af**

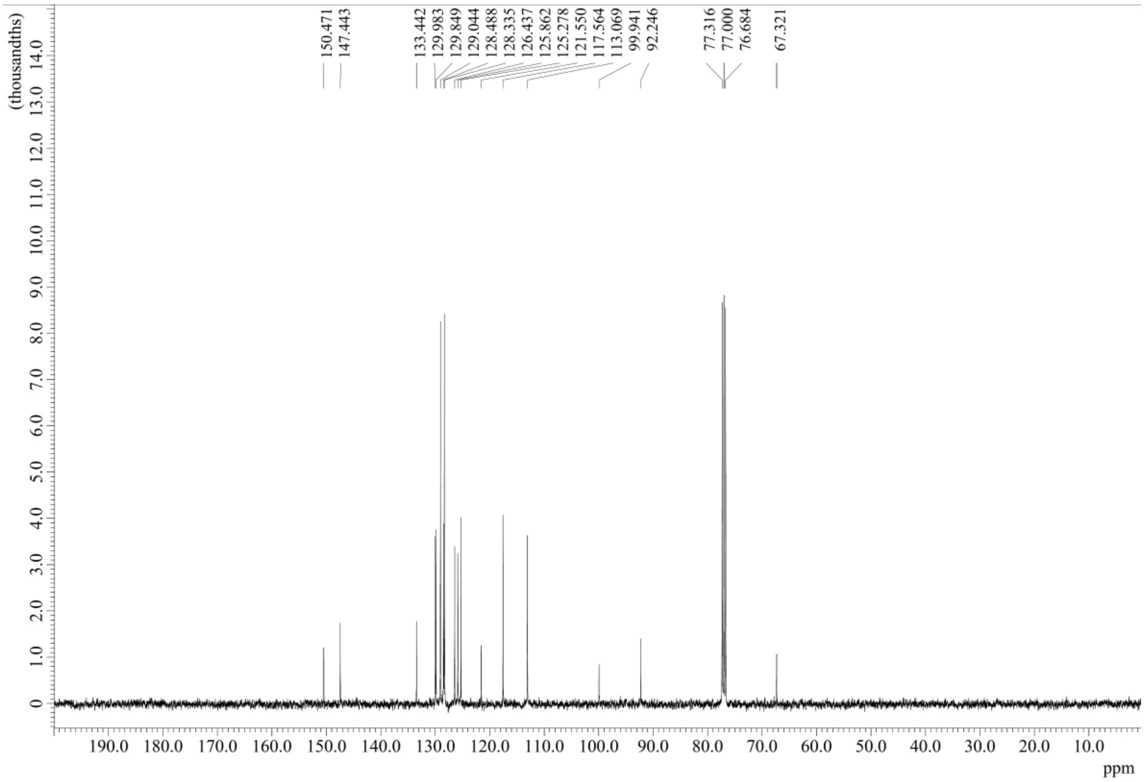

^{1}H NMR of **4ag**

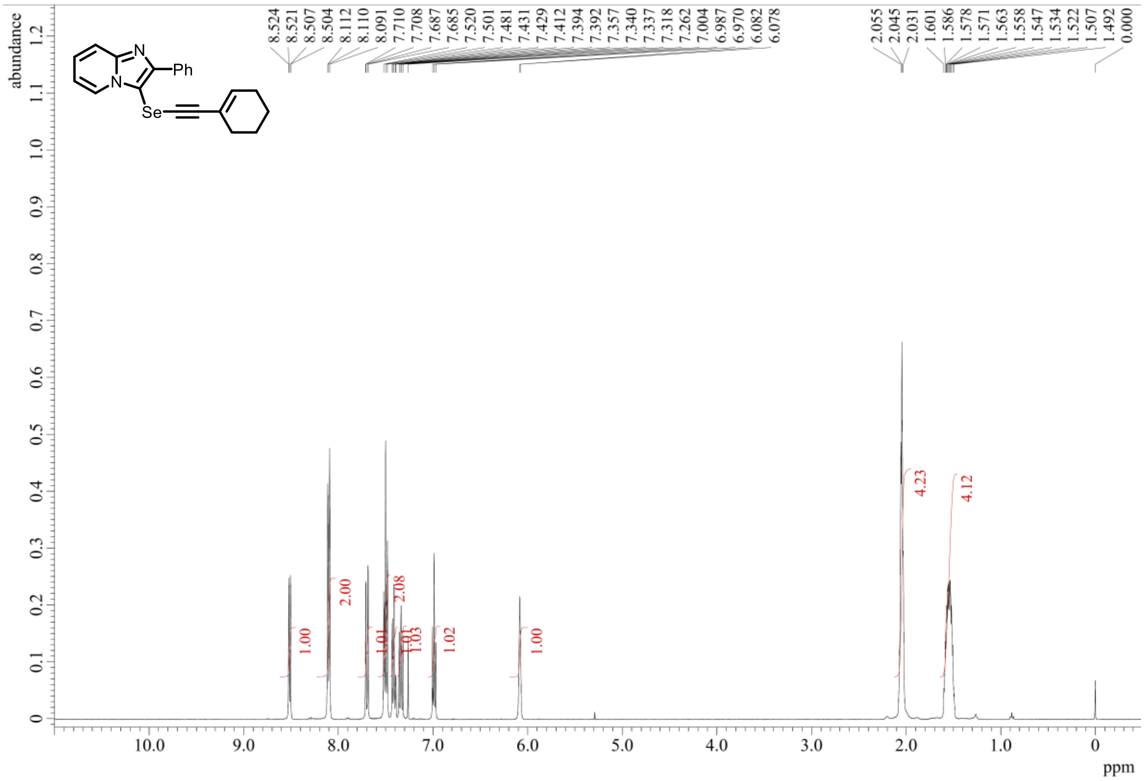

^{13}C NMR of **4ag**

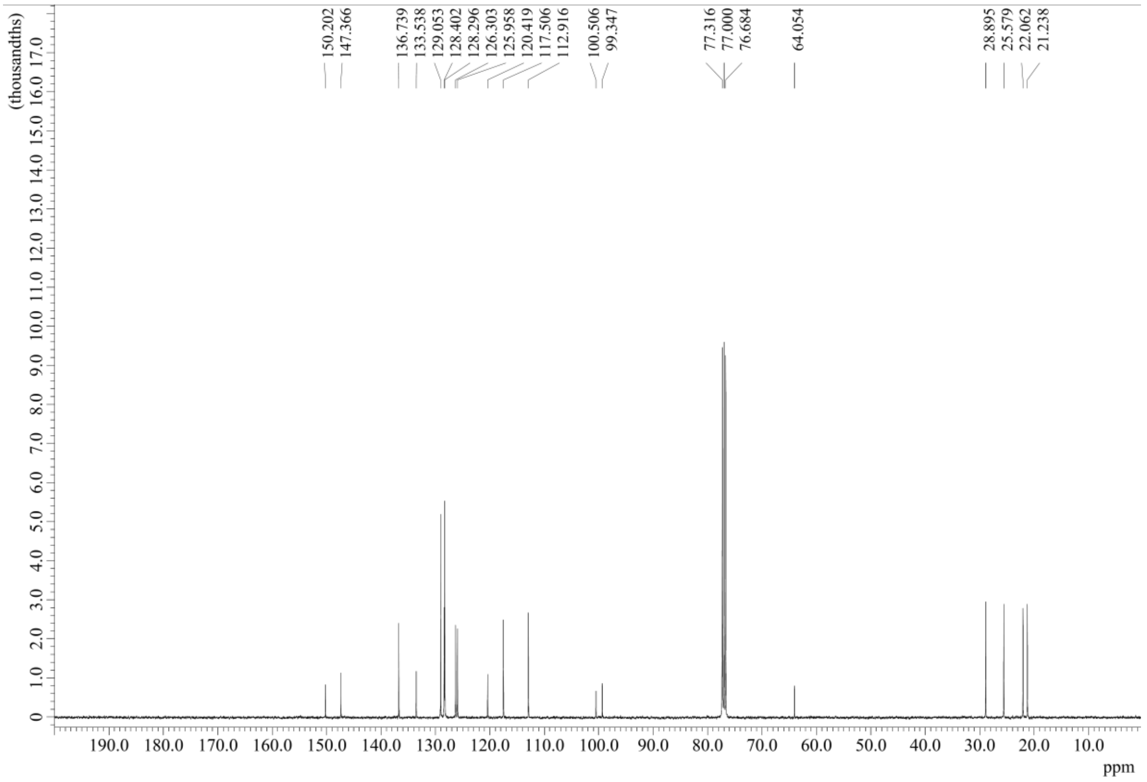

^{1}H NMR of **4ah**

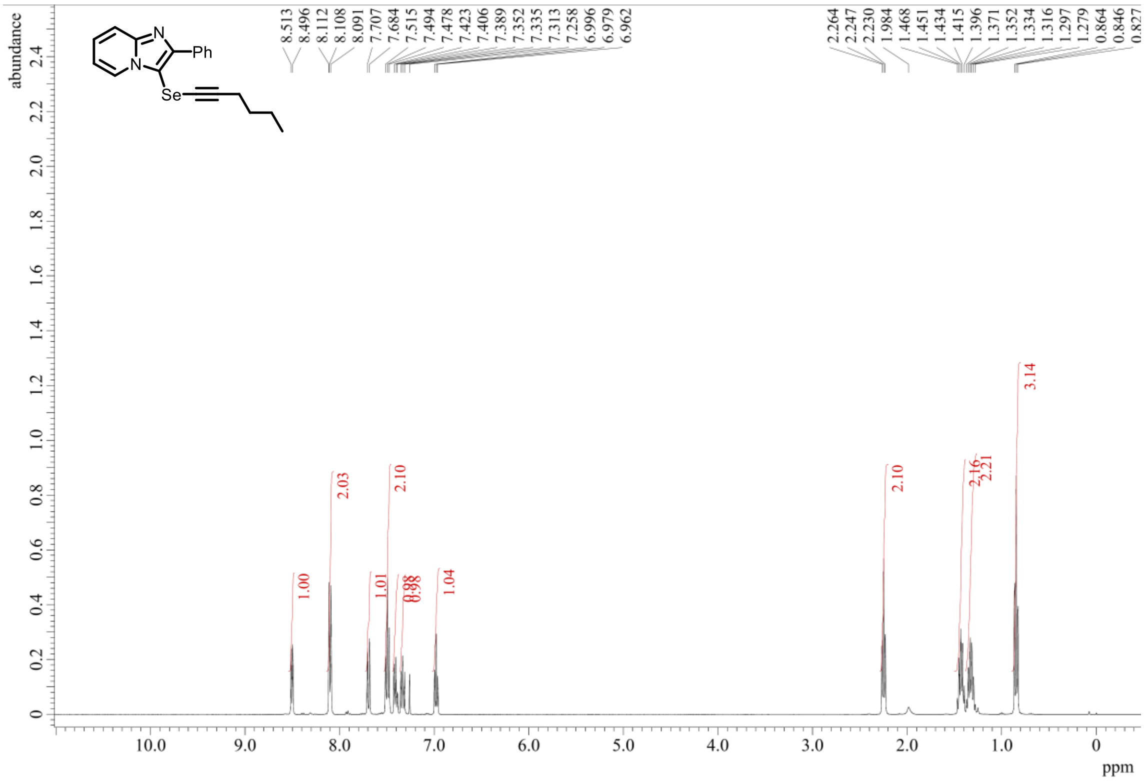

^{13}C NMR of **4ah**

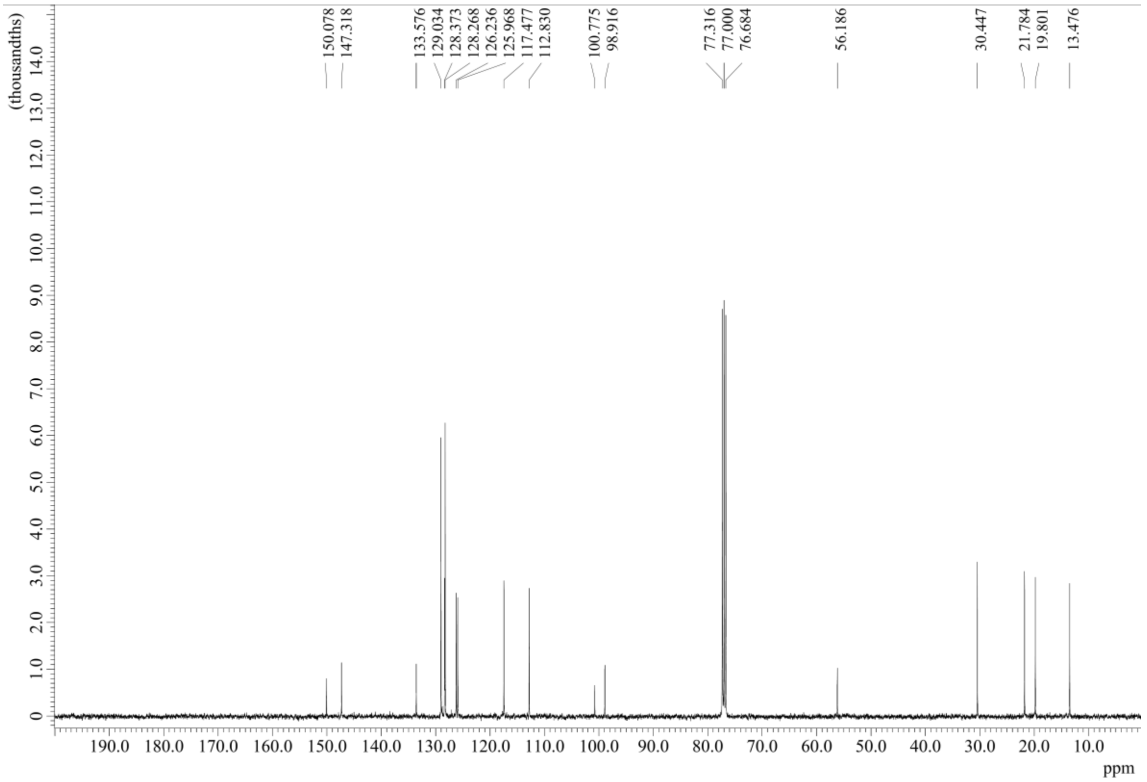

^{1}H NMR of **4ba**

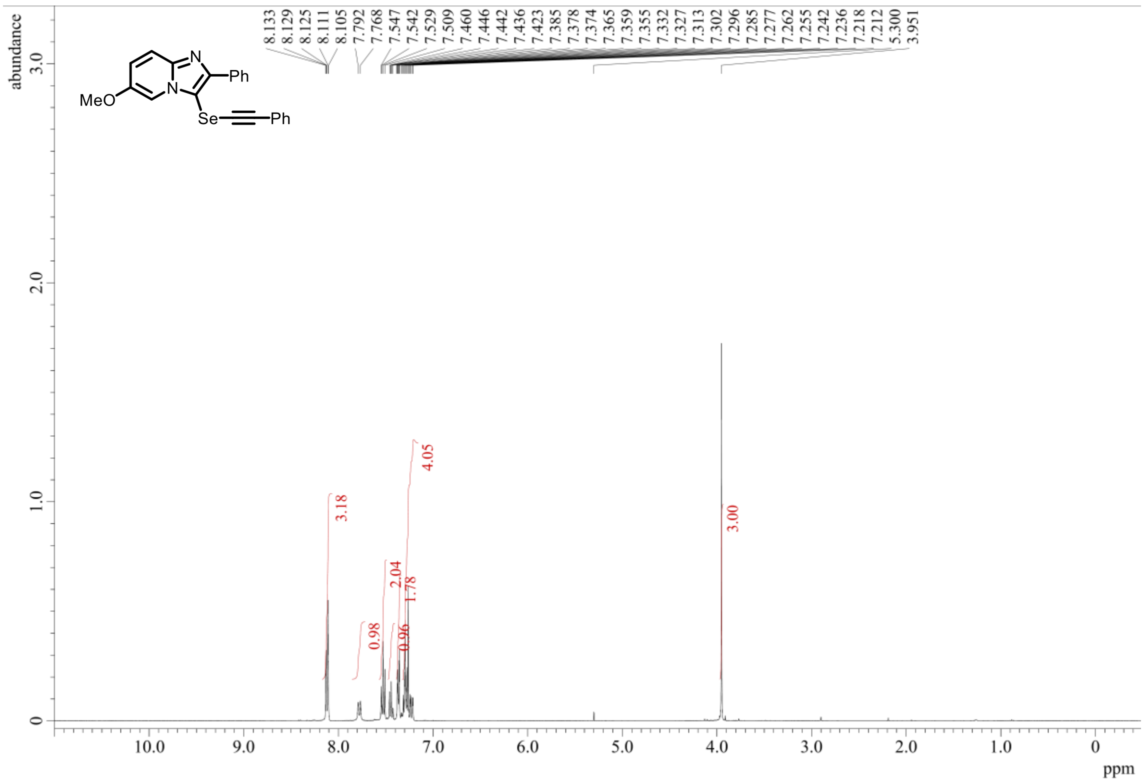

^{13}C NMR of **4ba**

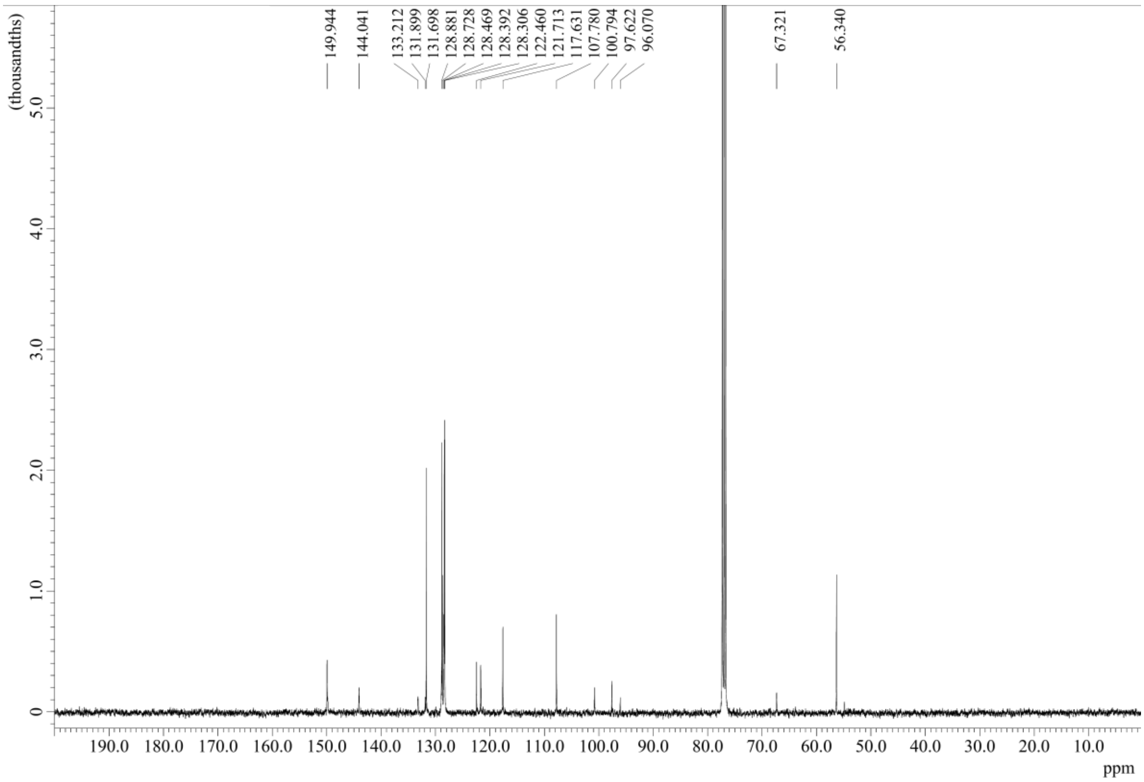

^{1}H NMR of **4ca**

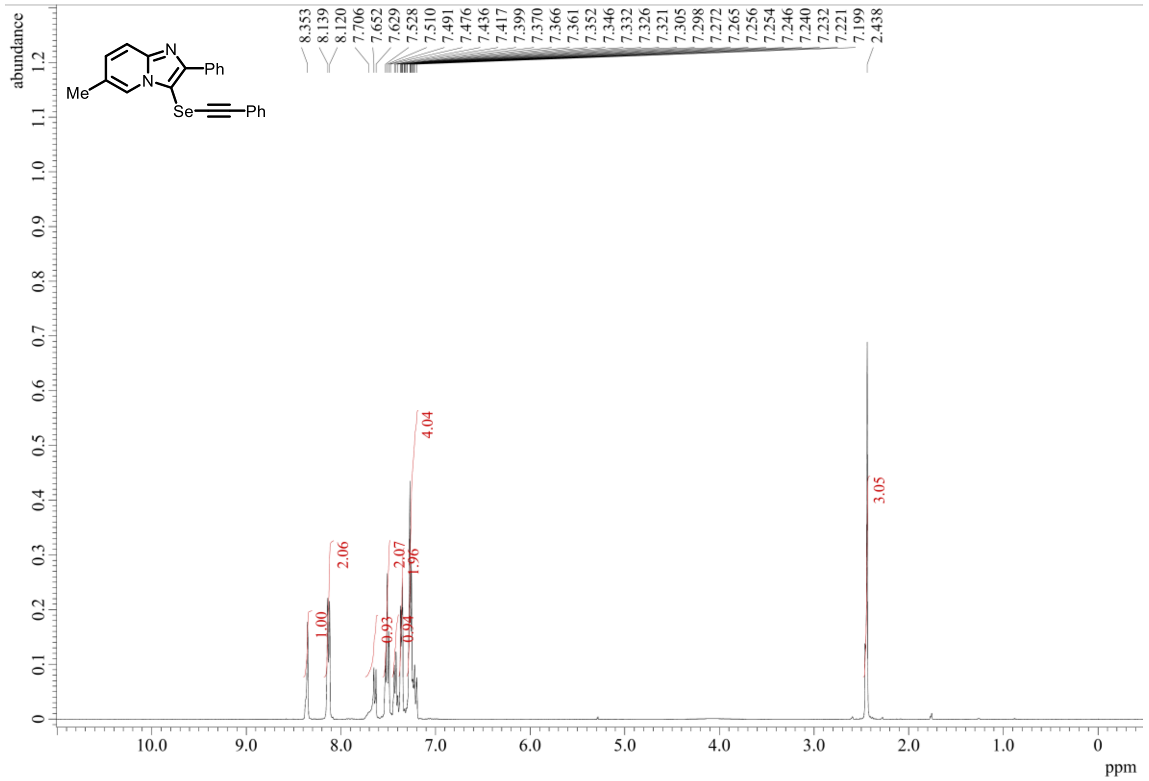

^{13}C NMR of **4ca**

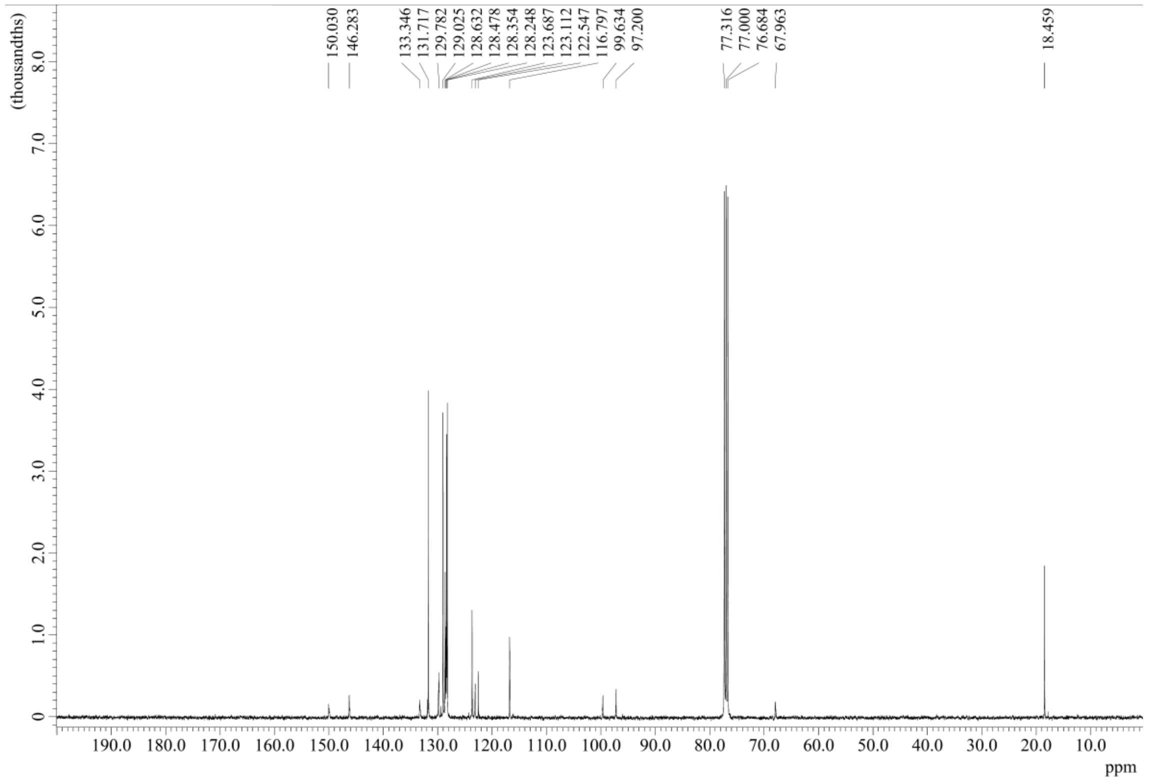

^{1}H NMR of **4da**

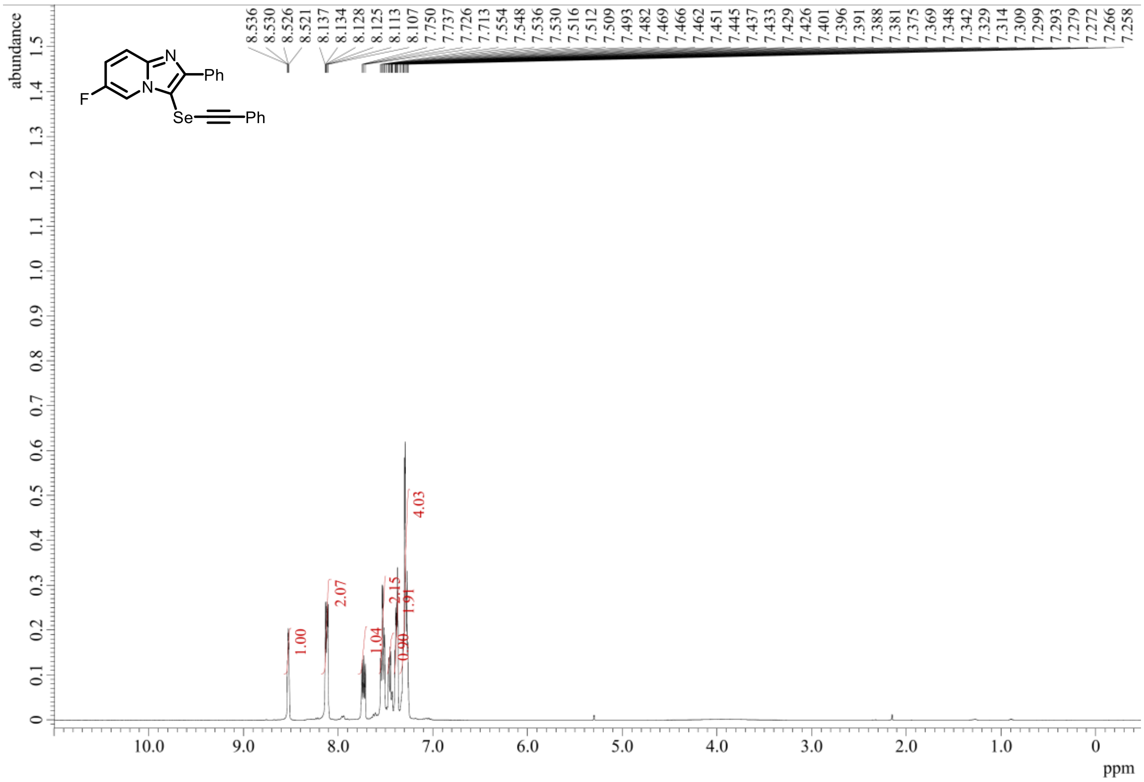

^{13}C NMR of **4da**

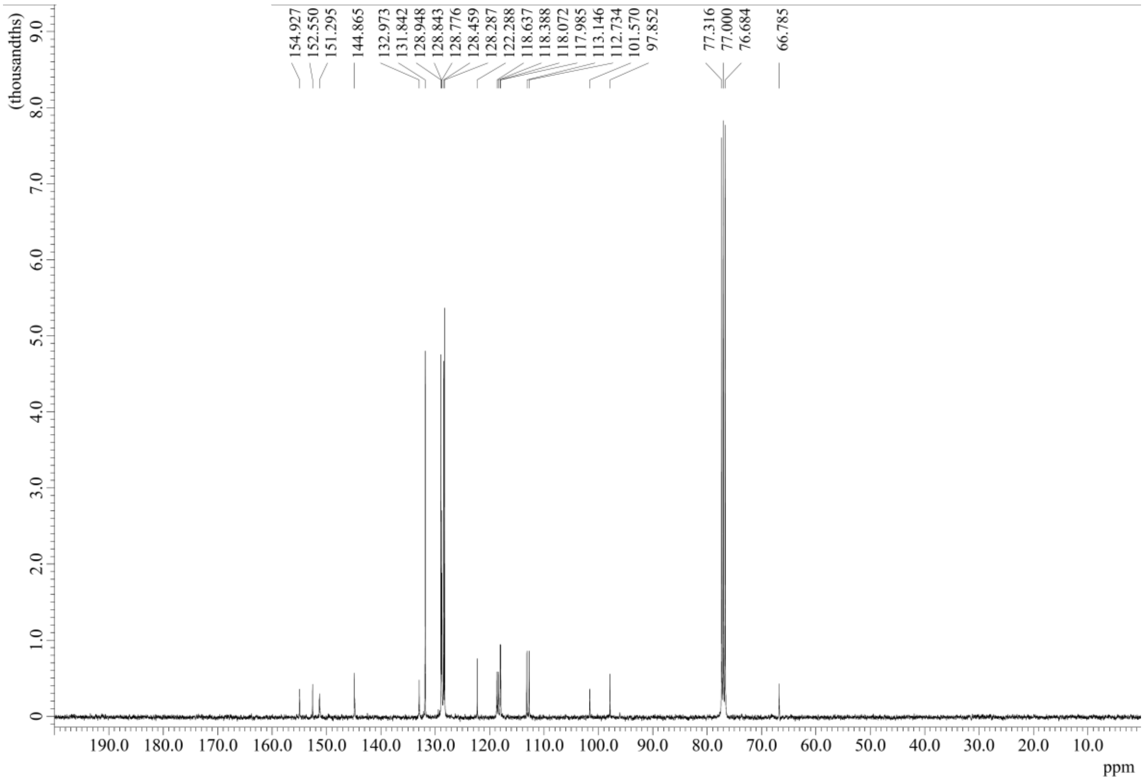

^{1}H NMR of **4ea**

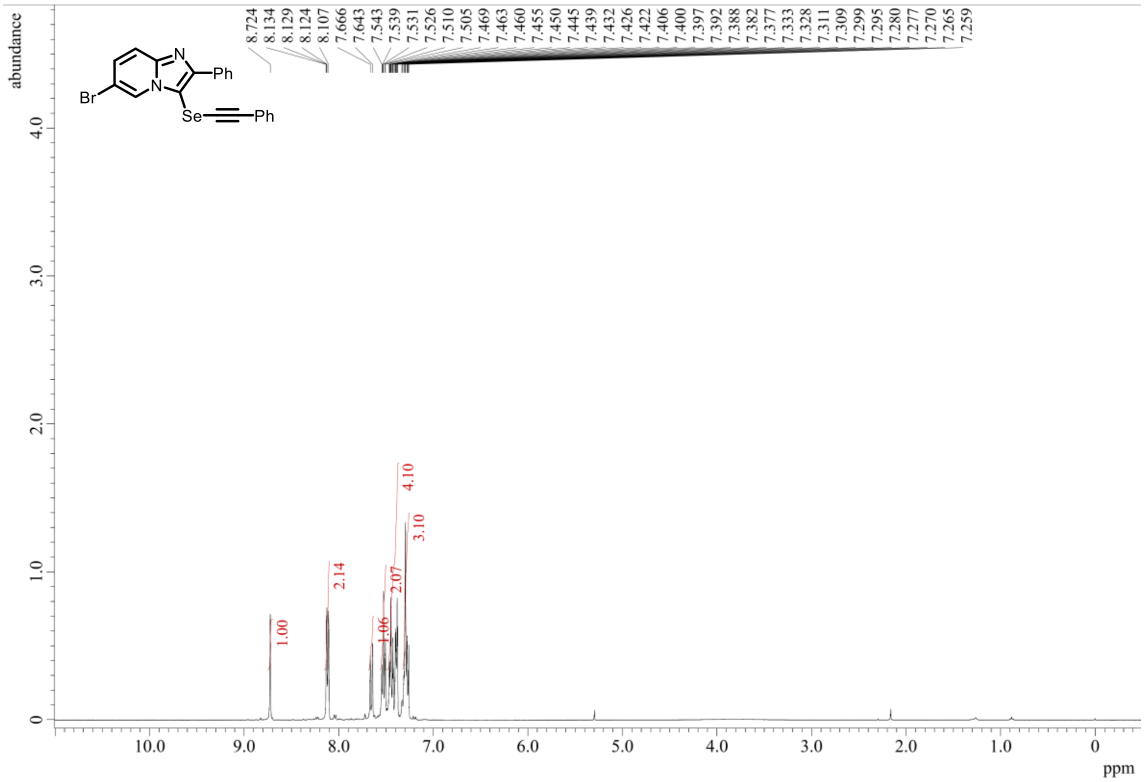

^{13}C NMR of **4ea**

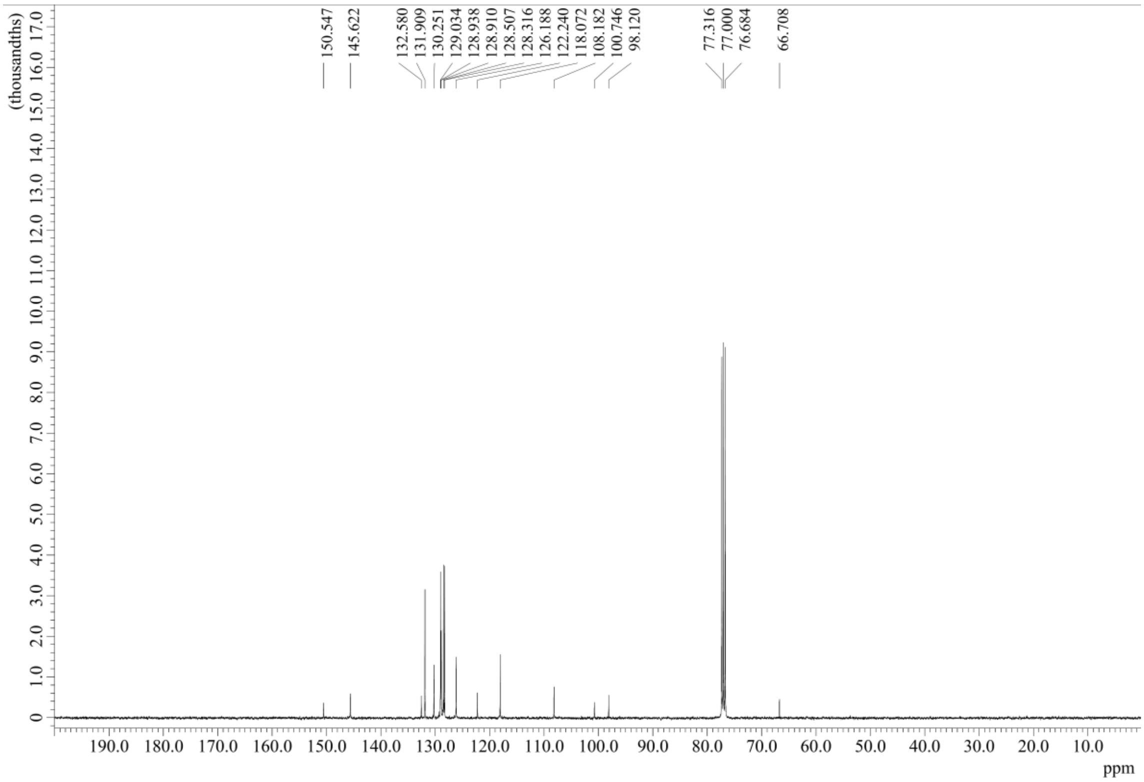

^{1}H NMR of **4ga**

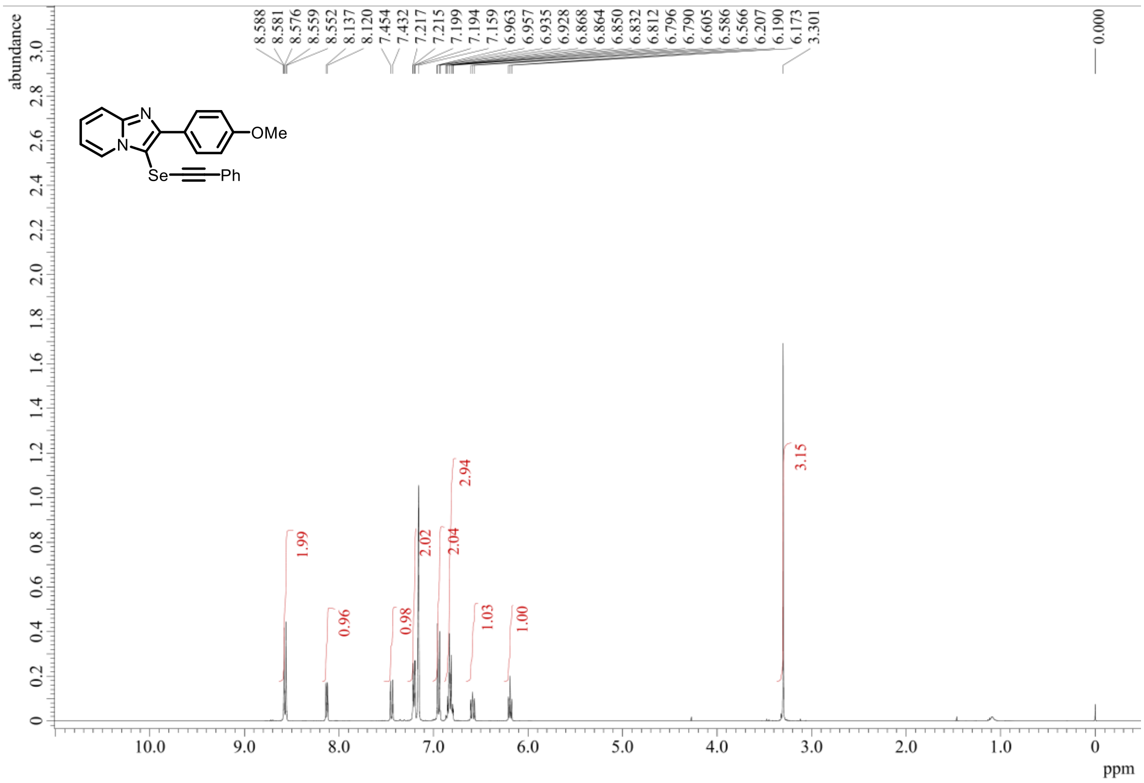

^{13}C NMR of **4ga**

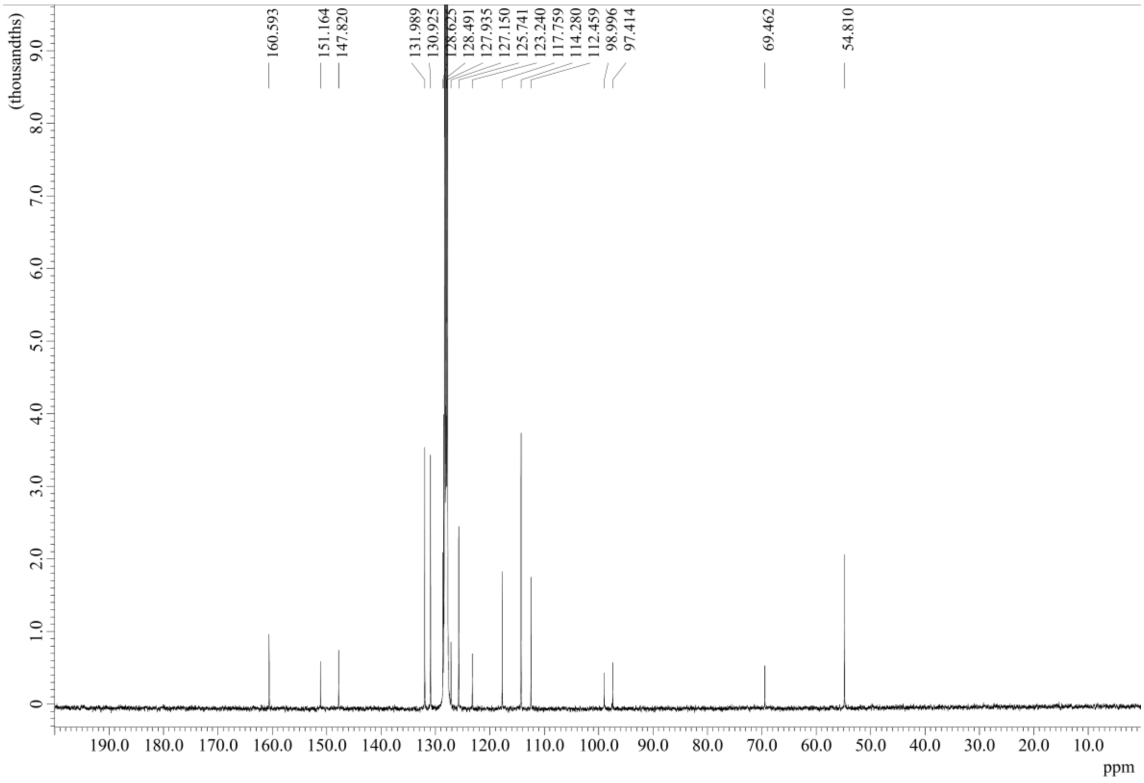

^{1}H NMR of **4ha**

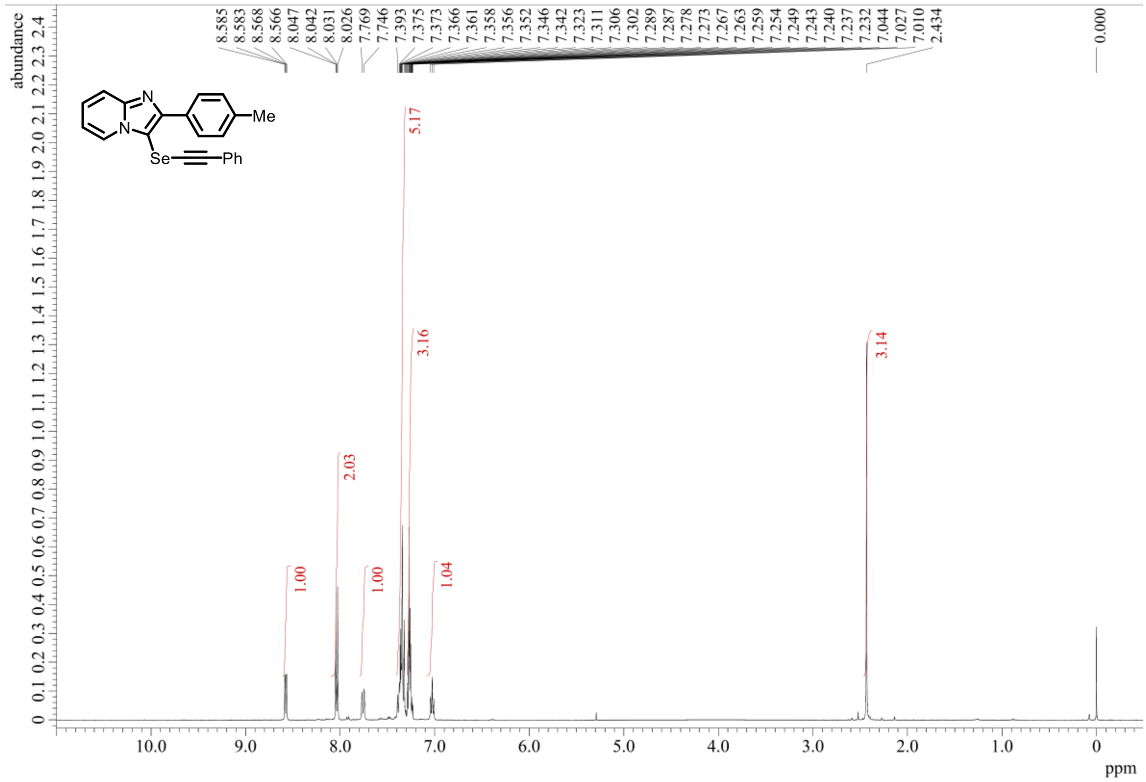

^{13}C NMR of **4ha**

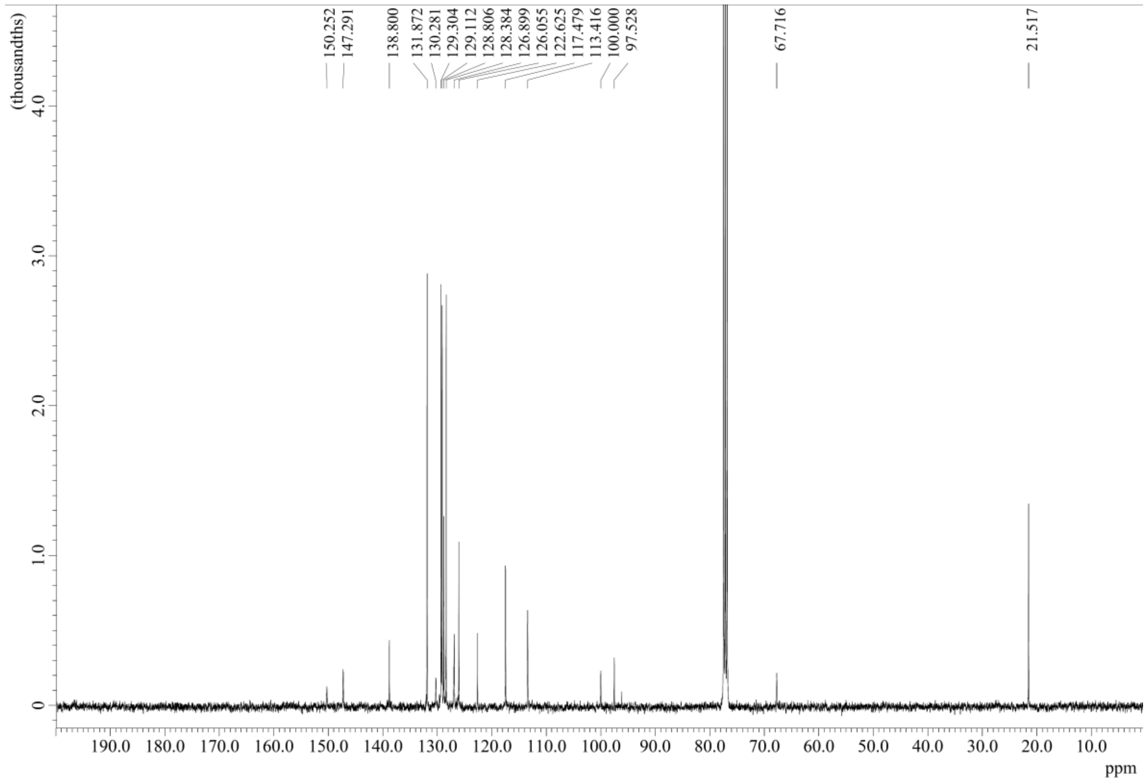

^{1}H NMR of **4ia**

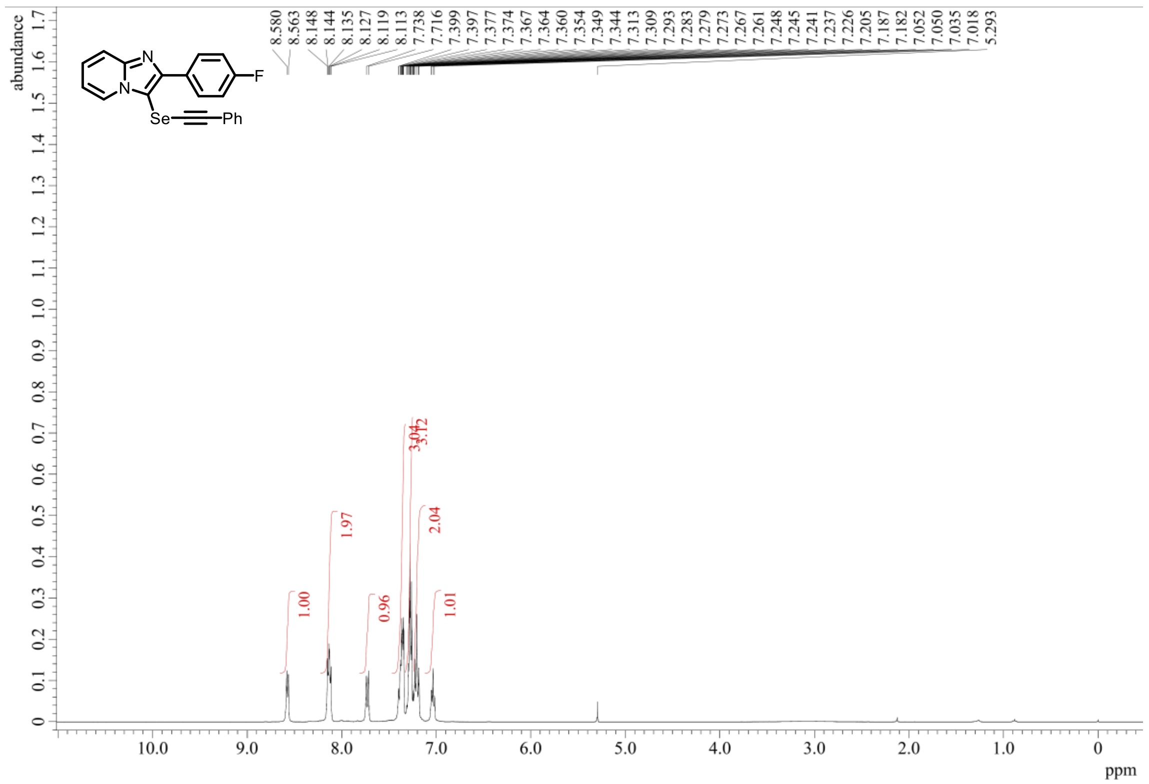

^{13}C NMR of **4ia**

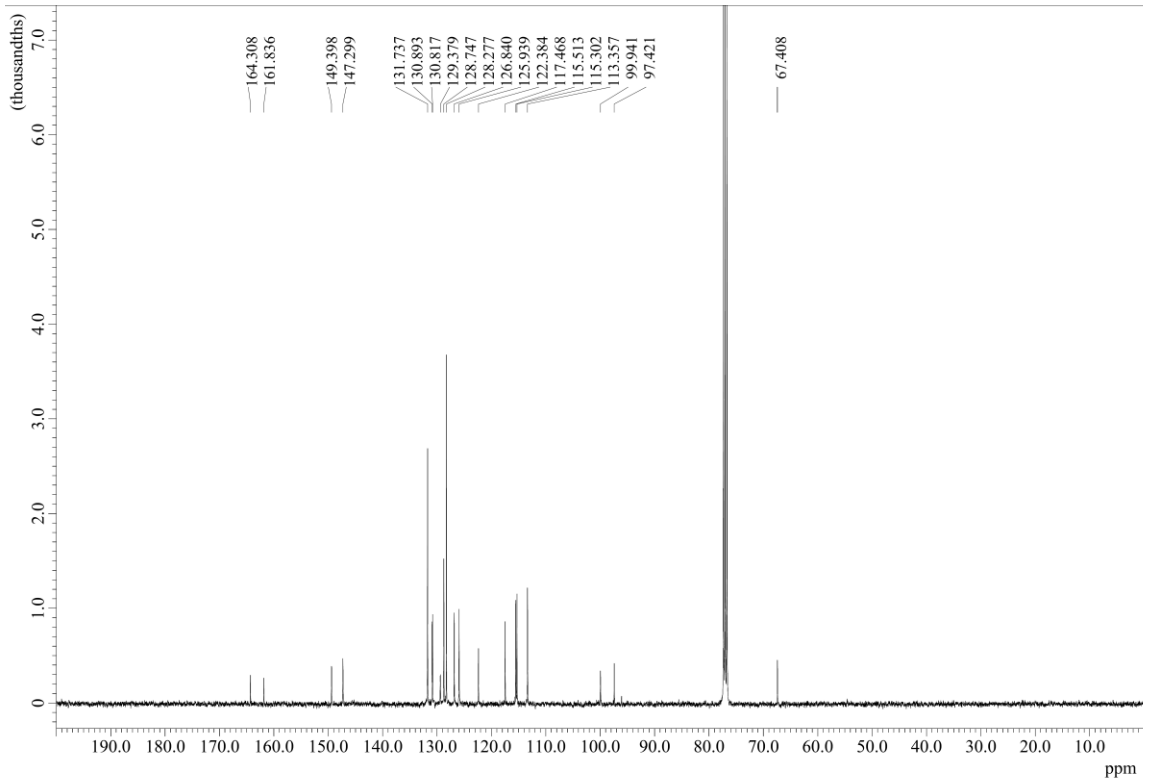

^{1}H NMR of **4ja**

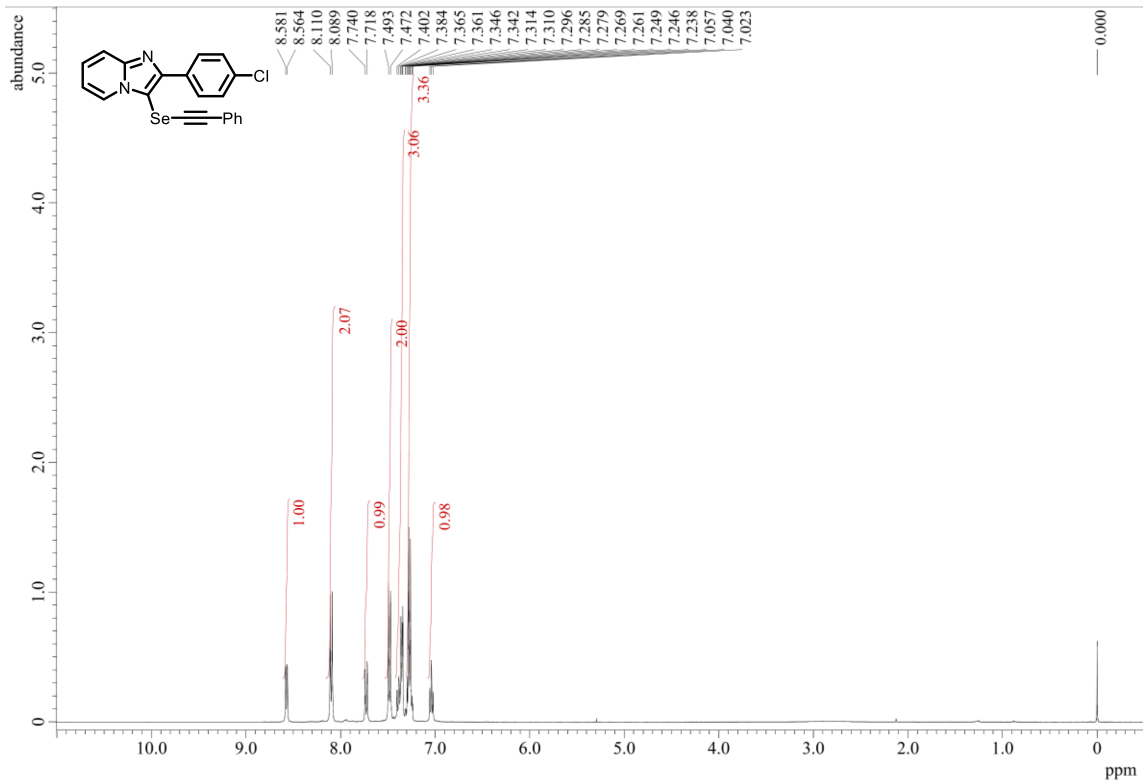

^{13}C NMR of **4ja**

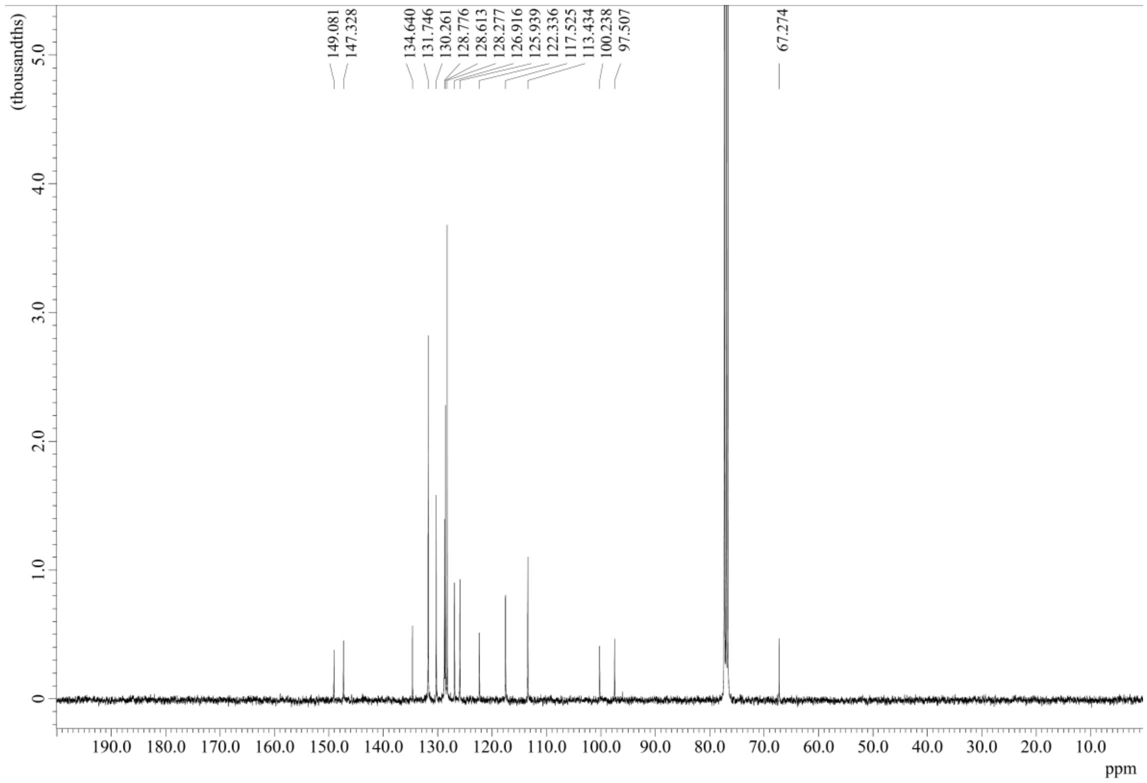

^{1}H NMR of **4ka**

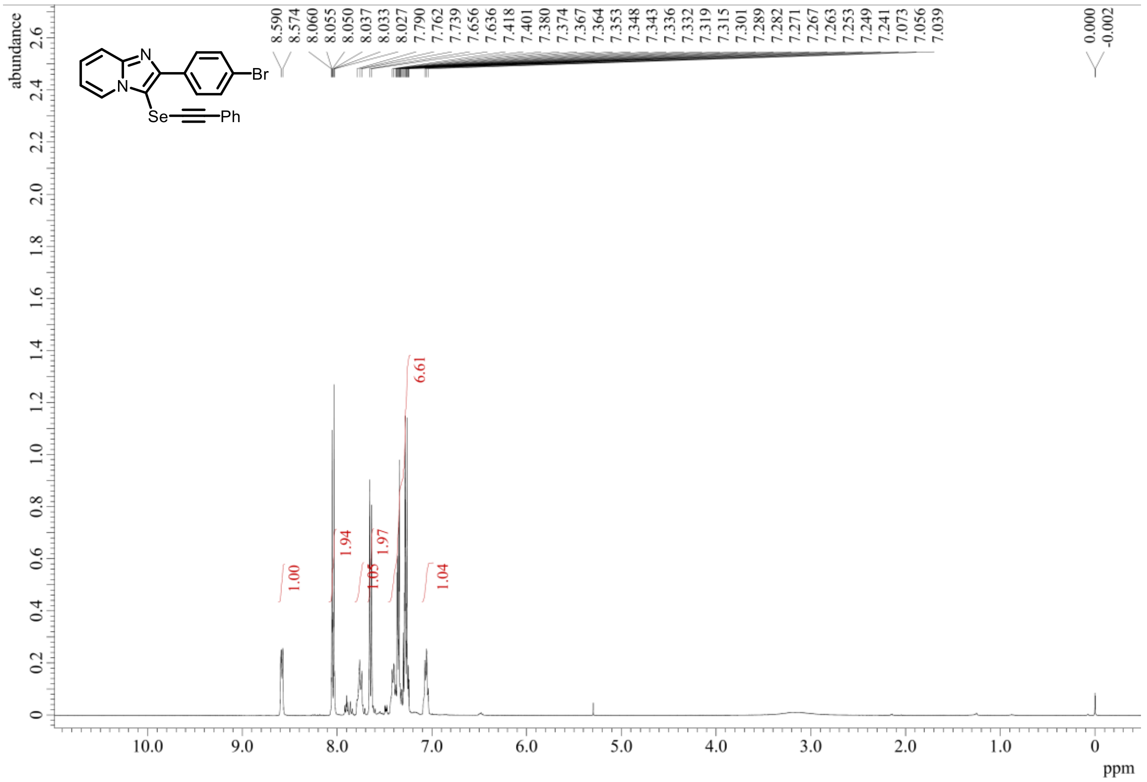

^{13}C NMR of **4ka**

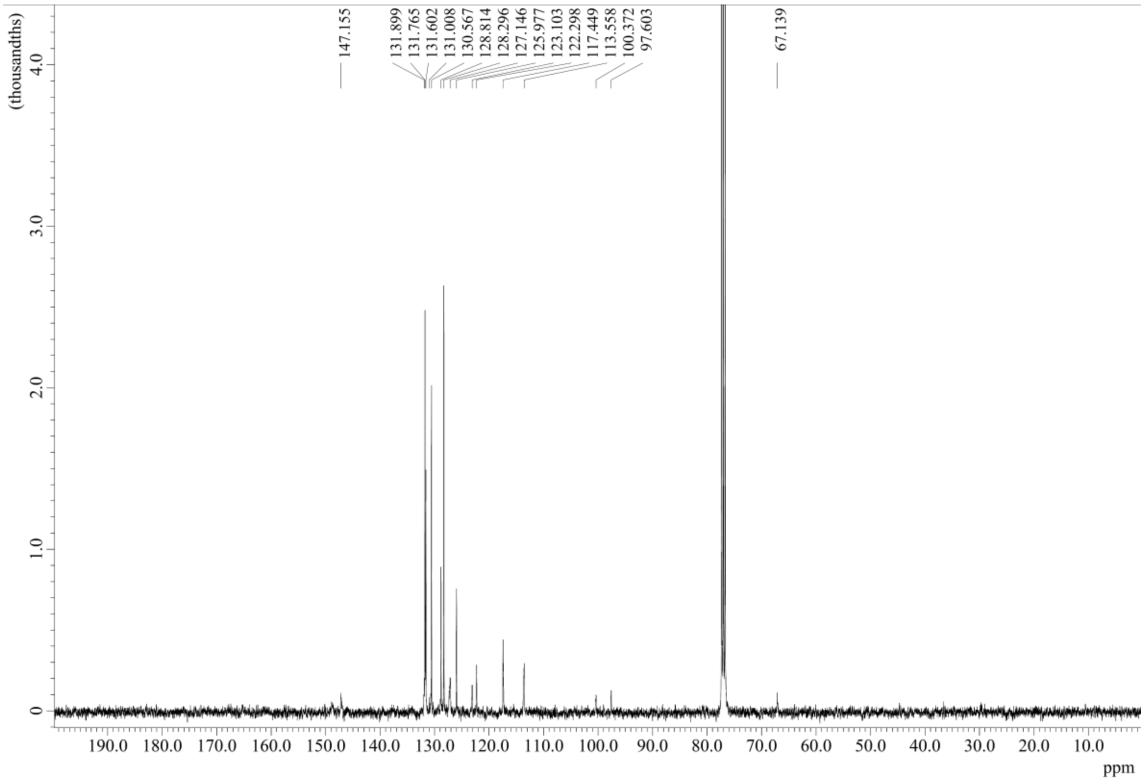

^{1}H NMR of **4la**

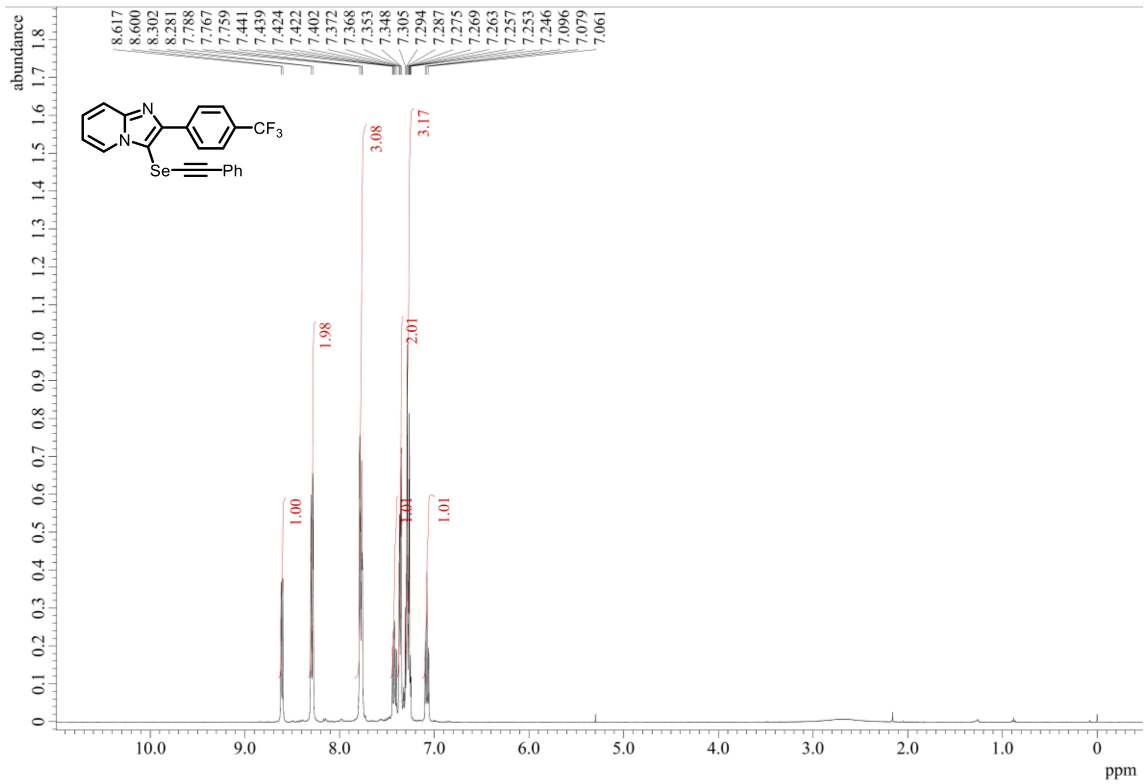

^{13}C NMR of **4la**

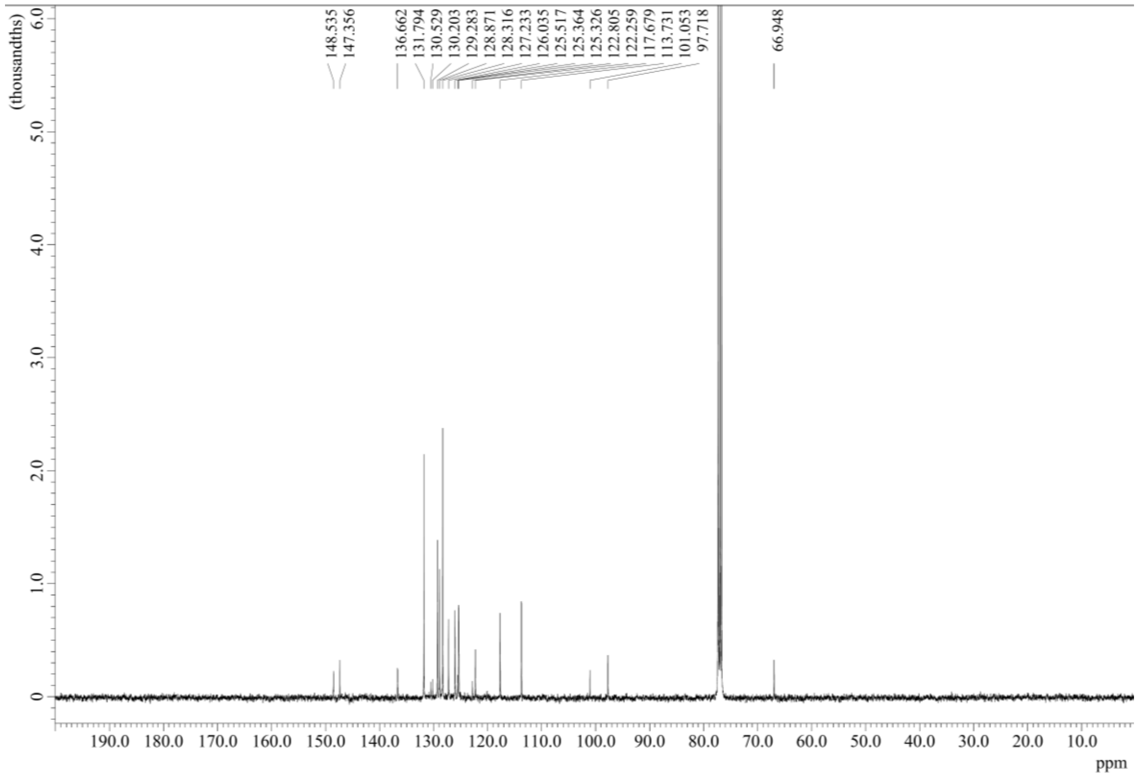

^{1}H NMR of **4ma**

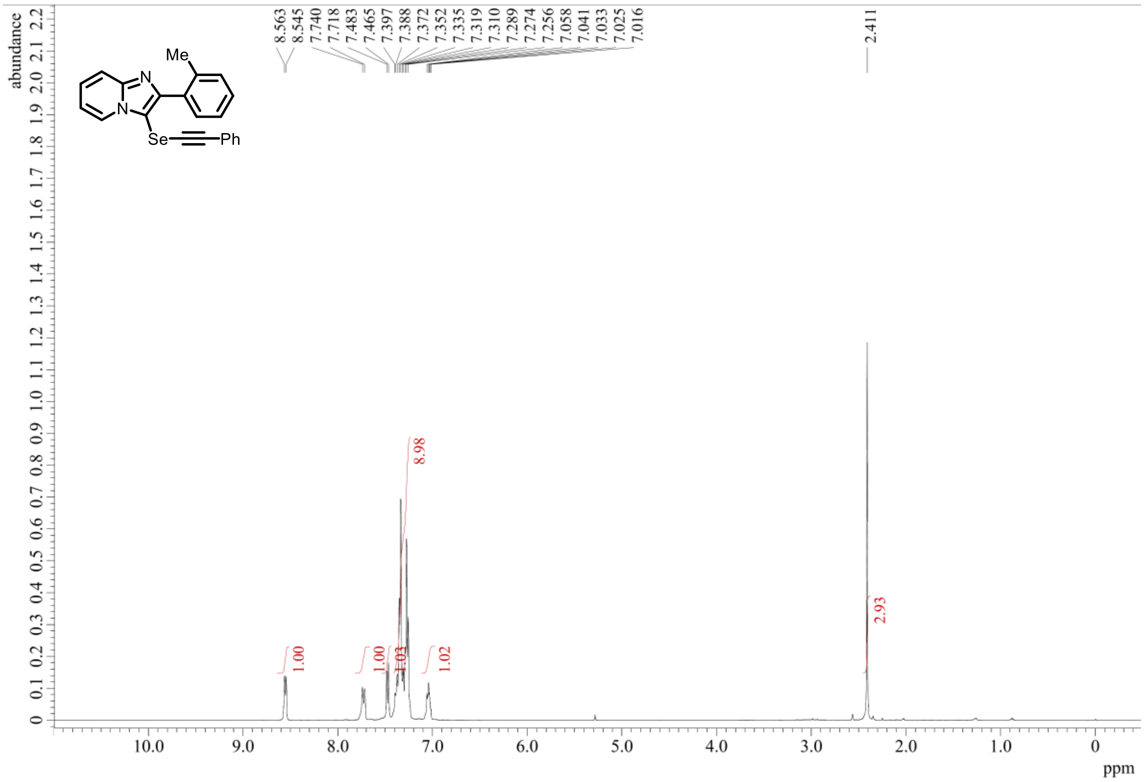

^{13}C NMR of **4ma**

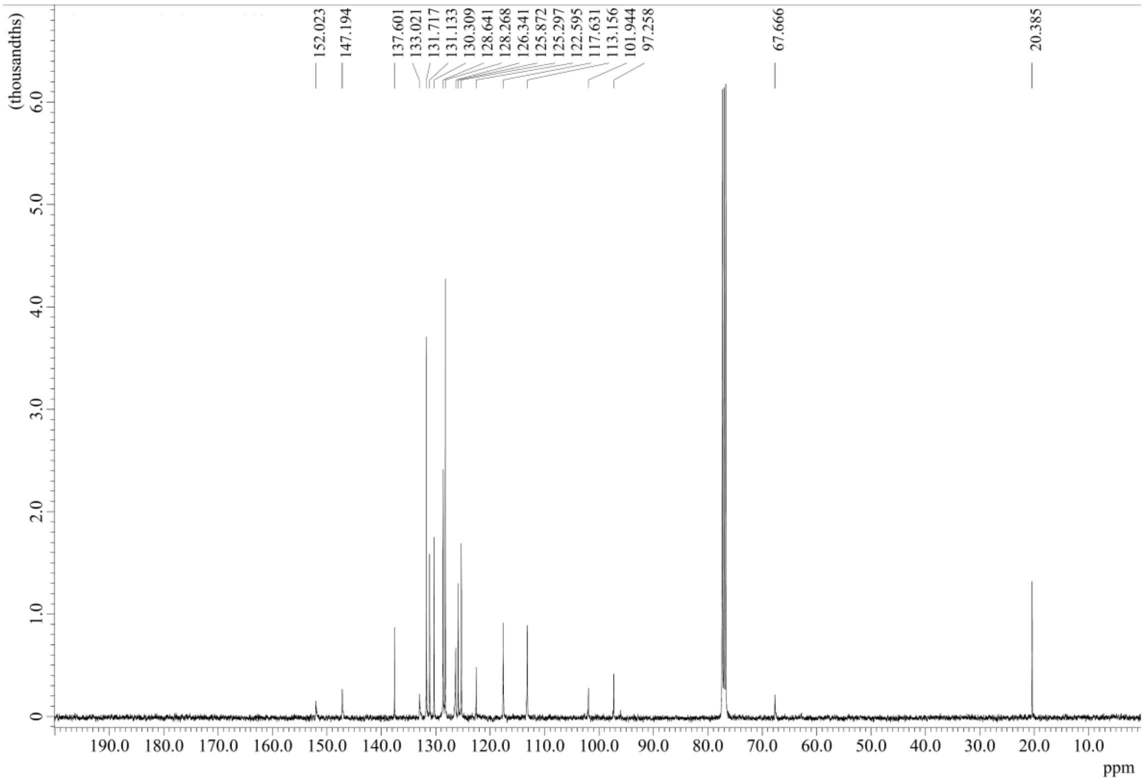

^{1}H NMR of **8**

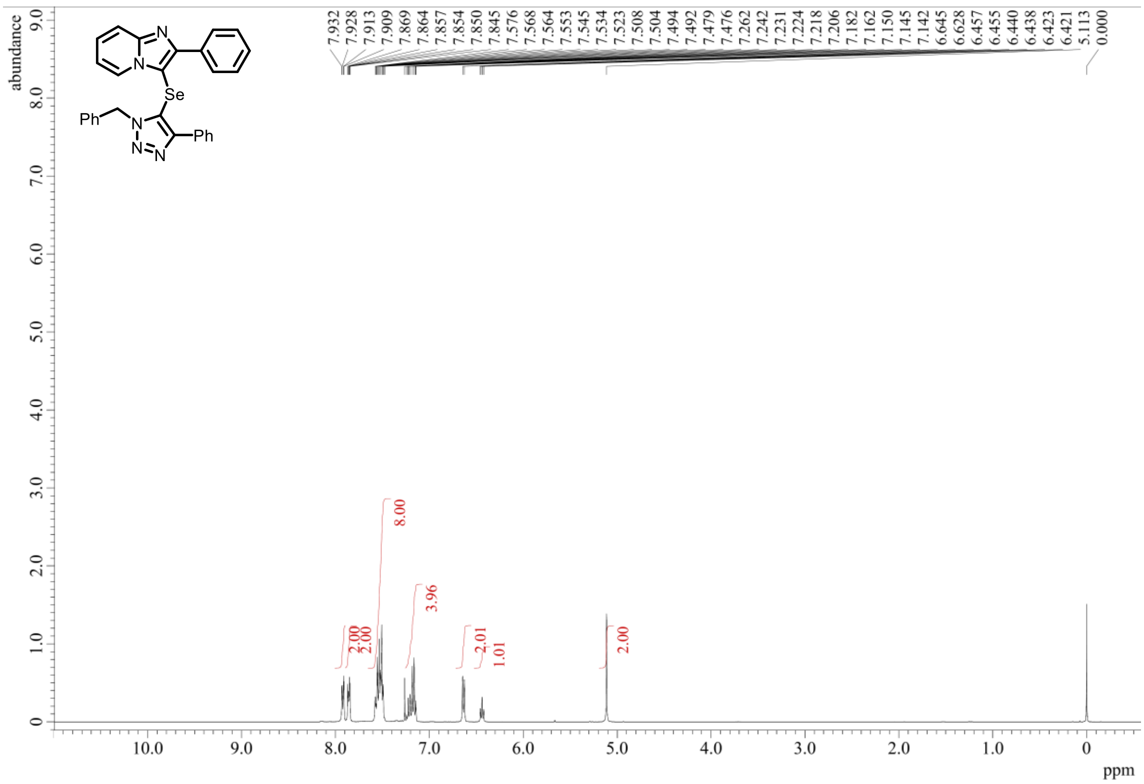

^{13}C NMR of **8**

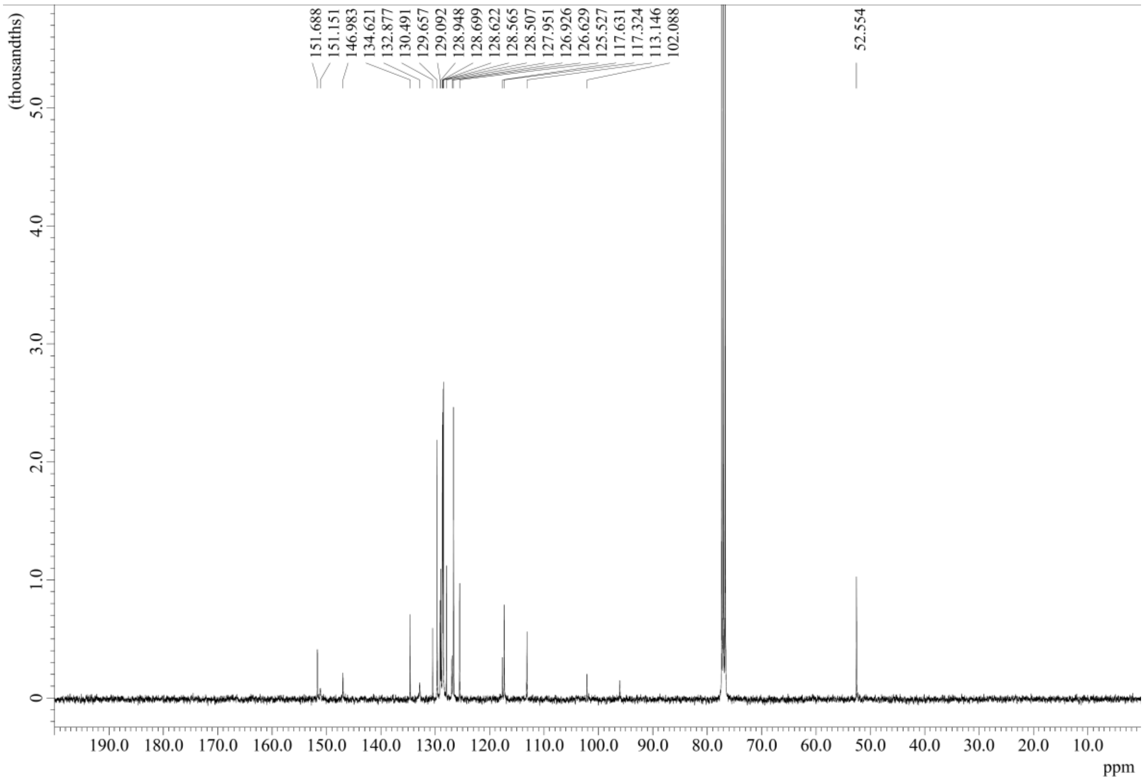

Supplement: File 1 — Characterization data of all new compounds, synthetic procedures for compounds 6–8, X-ray crystallography details, and copies of spectra. [file Beilstein_J_Org_Chem-18-863-s001.pdf]
